# Insights into Structure–Property Relationships in Quinoline–Based D–π–A Systems: A Synthetic and Computational Approach

**DOI:** 10.3390/molecules31203753

**Published:** 2026-10-19

**Authors:** Cristian Rojas-Peña, Roger Espinosa-Saez, Camilo Guzmán-Terán, Claudio A. Jiménez, Angie Ortega Villadiego, Jairo Enrique Salazar, Dayanna Orosco, Yeray A. Rodríguez-Núñez, Jhon J. López, Carlos Mario Meléndez, Efraín Polo-Cuadrado

**Affiliations:** 1 Laboratorio de Radicales Libres y Antioxidantes, Facultad de Ciencias Químicas y Farmacéuticas, Universidad de Chile, Av. Libertador Bernardo O’Higgins 1058, Santiago 8330111, Chile; cristian.rojas.p@ug.uchile.cl; 2 Grupo de Investigación IDEFARMA, Departamento de Regencia y Farmacia, Facultad Ciencias de la Salud, Universidad de Córdoba, Monteria 230002, Colombia; cantonioguzman@correo.unicordoba.edu.co; 3 Departamento de Química Orgánica, Facultad de Ciencias Químicas, Universidad de Concepción, Concepcion 4130000, Chile; cjimenez@udec.cl (C.A.J.); dorosco2026@udec.cl (D.O.); 4 Organic and Biomedical Chemistry Research Group, Molecular Biology Research Laboratory, Faculty of Basic Science, Universidad Del Atlantico, Barranquilla 081007, Colombia; angiecortega@mail.uniatlantico.edu.co (A.O.V.); jairosalazar@mail.uniatlantico.edu.co (J.E.S.); 5 Laboratorio de Síntesis y Reactividad de Compuestos Orgánicos, Facultad de Ciencias Exactas, Departamento de Ciencias Químicas, Universidad Andrés Bello, Santiago 8370146, Chile; yeray.rodriguez@unab.cl; 6 Departamento de Química Orgánica, Facultad de Química y de Farmacia, Escuela de Química, Pontificia Universidad Católica de Chile, Santiago 7820436, Chile; jjlopez@uc.cl

**Keywords:** quinoline derivatives, styrylquinoline, density functional theory (DFT), donor–π–acceptor systems, hyperpolarizability, excited–state properties, structure–property relationships

## Abstract

In this study, four quinoline–based chromophores (**EP01**–**EP04**) with distinct π–conjugated architectures were studied to understand how molecular connectivity influences their electronic structure and nonlinear optical properties. Experimental characterization was combined with DFT and TD–DFT calculations to relate molecular structure to UV–Vis absorption, frontier–orbital distribution, and calculated electronic response. **EP01**, which contains a vinylene bridge connecting quinoline to an alkoxy–substituted aromatic ring, exhibited the smallest HOMO–LUMO gap in the series (5.65 eV) and the most red–shifted experimental absorption in methanol (353 nm). In comparison, the principal absorption maximum of **EP02** occurred at 265 nm. The results for **EP03** and **EP04** showed that band position and intensity depend not only on the HOMO–LUMO gap but also on the orbitals involved in each transition. The calculated solvent effect on the most intense transitions was limited, with shifts of 2–7 nm between toluene and water. **EP01** also exhibited the largest calculated first and second hyperpolarizabilities, approximately 2.58 and 2.92 times those of **EP02**, respectively. Together, these findings show that connecting the aromatic fragments through a continuous π–pathway favors the optical response of these derivatives and provides structural guidance for designing new quinoline–based chromophores.

## 1. Introduction

Efficient electronic communication through π–conjugated molecular frameworks is a fundamental prerequisite for developing high–performance organic materials with tailored optoelectronic and nonlinear optical (NLO) properties, optical absorption, and excited–state behavior [1,2]. Consequently, understanding the structural factors that govern electronic communication has become a central objective in the rational design of functional organic chromophores. Among the numerous heteroaromatic platforms explored for these applications, quinoline derivatives have attracted considerable attention owing to their rigid aromatic framework, intrinsic electron–deficient character, and exceptional synthetic versatility, which enable fine modulation of their electronic structure through appropriate molecular engineering [3,4,5,6].

One of the most successful molecular design strategies for enhancing NLO performance relies on the construction of donor–π–acceptor (D–π–A) systems, in which electron–rich and electron–deficient fragments are connected through conjugated bridges that promote intramolecular charge transfer (ICT) [7]. Extensive experimental and theoretical studies have demonstrated that variations in the donor or acceptor strength, conjugation length, and molecular planarity can substantially influence the electronic and optical properties of these chromophores. Nevertheless, despite the remarkable progress achieved in the development of D–π–A materials, the relative contributions of the intrinsic donor–acceptor strength and efficiency of π–electronic connectivity to the overall electronic and nonlinear optical responses remain insufficiently understood. While donor and acceptor substituents define the local electronic environment, their ability to influence the global electronic structure ultimately depends on the efficiency with which these regions communicate through the conjugated molecular framework [8,9].

Styrylquinoline derivatives are attractive molecular models for exploring fundamental structure–property relationships [4,10]. The incorporation of a vinylene bridge between the quinoline nucleus and an aromatic ring extends the π–conjugated pathway, facilitating long–range electronic delocalization and enhancing intramolecular charge transfer. Previous studies have shown that structural modifications of styrylquinoline systems significantly affect their absorption spectra, photophysical behaviors, electronic distributions, and nonlinear optical properties. Likewise, functionalization through donor and acceptor substituents, photoresponsive structural motifs, polymer incorporation, and alkali–metal doping has proven effective for tuning their optoelectronic performance [3,4,5,6]. However, these investigations have predominantly focused on optimizing molecular properties through empirical structural modifications, whereas comparatively little attention has been devoted to establishing whether π–electronic connectivity represents the principal structural factor governing electronic delocalization and nonlinear optical performance [11].

Addressing this question requires molecular systems in which the influence of π–connectivity can be distinguished from the presence of electronically differentiated substituents in the molecule. This distinction is essential because molecules containing comparable donor and acceptor fragments may exhibit markedly different electronic organizations depending on the efficiency with which these regions communicate across the molecular framework. Therefore, understanding the role of molecular connectivity extends beyond interpreting the behavior of individual compounds and provides general design principles for the development of next–generation quinoline–based chromophores with enhanced optoelectronic performance [4,6,12].

In this context, **EP01**–**EP04** were selected to examine how different modes of π–connectivity and substitution influence the properties of quinoline–based chromophores. **EP01** contains a vinylene bridge that extends conjugation between an alkoxy–substituted aromatic ring and the quinoline core. **EP02** and **EP03** both contain a benzodioxole group directly attached to quinoline, but differ in their quinoline substituents: **EP02** bears a fluoro group and a nonconjugated propanol side chain, whereas **EP03** bears a methoxy group. **EP04**, a directly linked chlorophenyl–quinoline derivative, provides a further comparison without the vinylene bridge or benzodioxole fragment. Together, these structures allow us to assess how the extent and arrangement of conjugation, alongside substituent identity, affect the electron–density distribution, excited–state behavior, and nonlinear optical response.

We hypothesized that the π–electronic connectivity and substituent identity jointly govern the electronic and nonlinear optical properties of these chromophores. To examine this relationship, **EP01**–**EP04** were synthesized and characterized using NMR, FT–IR, UV–Vis spectroscopy, and single–crystal X–ray diffraction techniques. The electronic structures of the synthesized complexes were investigated using density functional theory (DFT) and time–dependent DFT (TD–DFT), including analyses of the frontier molecular orbitals, global and local reactivity descriptors, Fukui functions, spin–density distributions, electronic transitions, and solvent effects. We also calculated the dipole moments, polarizabilities, polarizability anisotropies, and first– and second–order hyperpolarizabilities. This combined experimental and computational approach enabled a comparison of the relationship between specific structural features and the electronic, spectroscopic, and calculated nonlinear optical properties of the four compounds.

## 2. Results and Discussion

### 2.1. Synthesis

The four quinoline derivatives, **EP01**–**EP04**, were synthesized using two complementary strategies that reflected the structural diversity of the final products (Table 1), as follows. **EP01** was obtained through the Perkin–type condensation of quinaldine with 4–ethoxy–3–methoxybenzaldehyde in acetic anhydride under heating, affording the corresponding styrylquinoline in 21% yield as brown crystals (m.p. 121–123 °C). Condensation of quinaldines with aromatic aldehydes in acetic anhydride is a well–established approach for the synthesis of 2–styrylquinoline derivatives, with reaction conditions and yields depending on the nature of the substrates and the reaction protocol [13,14]. The moderate yield obtained for **EP01** is consistent with the variable efficiency commonly observed for these condensations and may reflect the influence of the electronic properties of the aldehyde and reaction conditions. In contrast, **EP02**–**EP04** were obtained through a two–step protocol involving a BiCl_3_–catalyzed Mannich/Friedel–Crafts reaction, followed by aromatization. For **EP02**, condensation of 4–fluoroaniline with piperonal, a domino reaction with 3,4–dihydro–2*H*–pyran, and final MnO_2_ oxidation afforded the product in 53% yield (m.p. 135–136 °C). **EP03** and **EP04** were synthesized analogously using *N*–vinyl–2–pyrrolidone and elemental sulfur (S_8_) at 220–230 °C for aromatization, yielding 59% (m.p. 139–141 °C) and 51% (m.p. 164–166 °C) products, respectively. These yields are consistent with those reported for related BiCl_3_–catalyzed Povarov reactions, which typically afford 2–arylquinolines in the 54–62% range [15].

Structural characterization by IR, ^1^H, and ^13^C NMR spectroscopy confirmed the expected connectivity. For **EP01**, vinylene doublets (*J* ≈ 16 Hz) at 7.29–7.59 ppm established the E geometry, while the methoxy and ethoxy carbons appeared at 55.8 and 14.7/64.2 ppm, respectively (Figures S1 and S2, Supporting Information). For **EP02**, the benzodioxole O–CH_2_–O singlet appeared at 6.10 ppm, the propanol hydroxyl as a triplet at 4.48 ppm, and the C–F couplings confirmed fluorine substitution at position 6 (Figure S4, Supporting Information). For **EP03**, the methoxy and dioxole singlets appeared at 3.93 and 6.02 ppm, with the corresponding carbons at 55.5 and 101.2 ppm, respectively (Figure S6, Supporting Information). For **EP04**, the methyl singlet at 2.55 ppm and the methyl carbon at 21.6 ppm confirmed the 6–methyl substituent (Figure S8, Supporting Information).

X–ray diffraction revealed distinct solid–state architectures (Figures S10–S18, Supporting Information). **EP01** crystallizes in the orthorhombic P2_1_2_1_2_1_ space group and adopts a planar geometry that promotes π–π stacking along the b–axis (Figure S11, Supporting Information). **EP02** crystallizes in the monoclinic P2_1_/n space group with a three–dimensional conformation stabilized by O–H···N hydrogen bonds and π–π interactions (Figures S12–S14, Supporting Information). **EP03** crystallized in the triclinic P–1 space group. In the absence of a hydrogen–bond donor, its packing is governed by weak C(sp^3^)–H···N interactions and π–π stacking between benzodioxole rings (Figures S15 and S16, Supporting Information). **EP04** crystallizes in the monoclinic P2_1_/c space group and packs exclusively through π–π stacking, forming discrete centrosymmetric dimers in a herringbone arrangement (Figures S17 and S18, Supporting Information). Notably, these divergent packing motifs, despite the shared biaryl linkage in **EP02**–**EP04**, parallel the behavior reported for related quinoline–carboxamide systems, in which the interplanar angle between the amide group and the heterocyclic ring dictates the hydrogen–bonding network and supramolecular organization [16]. Similarly, studies on styrylquinolines have established that the substituent identity and molecular planarity control the prevalence of π–stacked dimers and infinite chains in the crystal lattice [17]. These comparisons reinforce that the solid–state organization of **EP01**–**EP04** is governed by the interplay between peripheral substitution and inherent rigidity of the quinoline core.

From a synthetic perspective, the Perkin route for **EP01** is operationally simple but affords lower yields, whereas the multistep routes for **EP02**–**EP04** provide superior overall yields and greater structural flexibility in accessing diverse 2–arylquinoline architectures.

### 2.2. Electronic Structure and Reactivity Descriptors

To characterize the electronic properties and reactivity of the **EP01**–**EP04** series, frontier molecular orbitals and global reactivity descriptors were calculated using density functional theory (DFT) at the M06–2X/def2–TZVPP level, with solvent effects incorporated through the SMD implicit solvation model for methanol. The calculated HOMO and LUMO energies, HOMO–LUMO energy gaps (ΔE_H–L_), ionization potentials (IP), electron affinities (EA), chemical hardness (η), and electrophilicity indices (ω) are summarized in Table 2, while the spatial distributions of the frontier molecular orbitals are shown in Figure 1 [18].

The frontier molecular orbital analysis revealed distinct electronic patterns across the four quinoline derivatives. **EP01** exhibited the highest HOMO energy (−6.82 eV) and a LUMO energy of −1.16 eV, resulting in the smallest HOMO–LUMO gap of the series (5.65 eV). As shown in Figure 1, both frontier orbitals are distributed over a substantial portion of the molecular framework, involving the quinoline core, vinylene bridge, and substituted aromatic ring. This distribution is consistent with the extended π–conjugated architecture of **EP01**, in which the 4–ethoxy–3–methoxyphenyl moiety is directly connected to the quinoline system through the vinylene spacer. Consequently, the alkoxy–substituted aromatic fragment is not electronically isolated from the heteroaromatic core but forms part of a continuous conjugated pathway. The combination of this extended conjugation with the electron–donating alkoxy substituents provides a reasonable structural basis for the comparatively small frontier–orbital separation observed for **EP01**.

In **EP02**, the frontier orbital distribution differed significantly from that of **EP01**. Although the HOMO and LUMO involve regions of the quinoline and benzo[*d*][1,3]dioxole π–systems, their distribution is less extensive across the entire molecular structure. In particular, the propan–1–ol substituent attached to the quinoline ring does not provide an additional conjugated pathway and contributes little to the frontier π–orbitals. This structural organization is accompanied by a relatively stable HOMO (−7.31 eV) and the highest–energy LUMO of the series (−0.85 eV), producing a large ΔE_H–L_ value (6.46 eV). Thus, despite the presence of the electronically active benzodioxole fragment, **EP02** exhibited a substantially larger frontier orbital separation than **EP01** and **EP03**, although it was slightly smaller than that calculated for **EP04**.

**EP03**, which also contains a benzo[d][1,3]dioxole fragment but bears a methoxy substituent at the quinoline core and lacks the propan–1–ol side chain present in **EP02**, displayed an intermediate electronic profile. Its HOMO (−6.93 eV) and LUMO (−0.92 eV) produce a ΔE_H–L_ of 6.01 eV. Figure 1 shows that the frontier orbitals are mainly distributed over the aromatic and heteroaromatic π–framework, with contributions from both quinoline and benzodioxole. The higher HOMO energy of **EP03** relative to that of **EP02** is consistent with the structural differences between these compounds, particularly the presence of the methoxy substituent on the quinoline framework. Nevertheless, its LUMO remains relatively high in energy, and consequently, its HOMO–LUMO gap remains larger than that calculated for **EP01**.

A different electronic pattern was observed for **EP04**, a structurally simpler 2–(4–chlorophenyl)–6–methylquinoline derivative. Its HOMO and LUMO are predominantly distributed over the conjugated quinoline–phenyl framework, which is consistent with the direct connection between the two aromatic units. **EP04** exhibited the lowest HOMO energy in the series (−7.48 eV), together with a LUMO energy of −0.98 eV, resulting in the largest HOMO–LUMO gap (6.50 eV). Thus, despite the direct conjugation between the quinoline and phenyl rings, **EP04** displayed a larger frontier–orbital separation than the other derivatives. In particular, the absence of the extended vinylene–linked donor–substituted aromatic system present in **EP01** is consistent with its more strongly stabilized HOMO and wider frontier orbital separation. Overall, the calculated gaps followed the order **EP01** (5.65 eV) < **EP03** (6.01 eV) < **EP02** (6.46 eV) < **EP04** (6.50 eV), indicating that direct aromatic connectivity alone does not necessarily produce a reduced frontier–orbital gap; rather, the electronic nature of the substituents and their integration into the conjugated framework are also important determinants of the frontier–orbital energies.

Global reactivity descriptors provided additional insights into the differences between the two catalysts. **EP01** exhibited the lowest chemical hardness (η = 1.49 eV) and the highest electrophilicity index (ω = 5.38 eV), consistent with its smaller frontier orbital gap, indicating a comparatively greater ability to reorganize the electron density in response to electronic perturbations. In contrast, **EP02** and **EP03** exhibited similar hardness values (1.71 and 1.69 eV, respectively) and nearly identical electrophilicity indices (4.76 and 4.75 eV, respectively), despite their structural differences. This similarity suggests that the modification of the substituents around the benzodioxole–quinoline framework affects the individual frontier orbital energies more strongly than the overall global electrophilic character of these two molecules [19,20].

**EP04** presents a distinctly harder electronic profile. It exhibits the highest ionization potential (IP = 6.18 eV) and electron affinity (EA = 2.55 eV) in the series, together with the highest chemical hardness (η = 1.82 eV). The high IP is consistent with the strongly stabilized HOMO and indicates a comparatively greater energy cost for electron removal. Interestingly, although **EP04** also has the highest EA, its LUMO (−0.98 eV) is not the lowest in energy among the four compounds, illustrating that the adiabatic electron affinity cannot be interpreted solely from the neutral–state LUMO energy because it also incorporates the electronic and structural relaxation of the anionic state. Its electrophilicity index (ω = 5.25 eV) is higher than those of **EP02** (4.76 eV) and **EP03** (4.75 eV) and approaches that of **EP01** (5.38 eV), reflecting the combined effect of its high electronegativity (χ = 4.37 eV) and electron affinity. Taken together, these descriptors characterize **EP04** as the electronically hardest member of the series and emphasize that frontier orbital energies and adiabatic global reactivity descriptors provide complementary, rather than interchangeable, information.

Overall, the electronic properties of **EP01**–**EP04** reflect the interplay between the substituent effects and their integration into the conjugated molecular framework. **EP01** displayed the most distinctive profile, combining an extended styrylquinoline π–system with the smallest ΔE_H–L_ and lowest chemical hardness. **EP02** and **EP03** exhibited intermediate frontier orbital gaps and similar global electrophilicity indices, despite the differences in substitution around the quinoline framework. In contrast, **EP04** displayed the largest ΔE_H–L_ and highest chemical hardness of the series, together with the most stabilized HOMO and highest ionization potential. These results show that direct π–connectivity between aromatic units is not sufficient to minimize the frontier–orbital separation. Rather, the extent and topology of conjugation, together with the electronic nature and position of the substituents, collectively determine the frontier orbital energies and global electronic properties of quinoline derivatives [18,19,20].

To further characterize the spatial distribution of the electronic response across the **EP01**–**EP04** series, condensed Fukui functions (*f*^+^ and *f*^−^) and spin densities of the corresponding radical anions (RA) and radical cations (RC) were calculated at the M06–2X/def2–TZVPP level using the SMD implicit solvation model in methanol. The condensed values are provided in Tables S1–S4 of the Supporting Information, while the corresponding Fukui and spin–density isosurfaces are shown in Figure 2, Figure 3 and Figure 4. For clarity, the discussion of the condensed descriptors is restricted to atomic contributions of 0.10 or greater, allowing the most electronically relevant sites to be identified without overinterpreting minor contributions. The Fukui functions are a central tool in conceptual DFT for locating reactive sites. The *f*^+^ function is associated with regions where the molecule tends to accept electron density; therefore, it allows the identification of the most susceptible sites for reduction processes or nucleophilic attack. In contrast, the *f*-*function* indicates the regions from which electron density can be more easily removed and is therefore related to zones that are more prone to oxidation or electrophilic attack. Thus, while *f*^+^ informs the local ability to accept electrons, *f*^−^ describes the local ability to donate electron density.

The Fukui analysis reveals different patterns of local reactivity across the series. For **EP01**, the only condensed *f*^+^ value reaching the selected threshold is located at C12 (*f*^+^ = 0.10), situated within the vinylene bridge connecting the quinoline and alkoxy–substituted aromatic fragments, whereas no individual *f*^−^ value reaches 0.10. Nevertheless, the Fukui isosurface (Figure 2) shows that the electronic response is distributed across several regions of the conjugated framework rather than being confined to a single atomic center. This behavior is consistent with the extended π–conjugated architecture of **EP01**, for which the local response is distributed over multiple atoms, and therefore, individual condensed Fukui contributions remain relatively moderate.

A different distribution was observed for **EP02**. No individual *f*^+^ value reached 0.10, whereas O23 exhibited *f*^−^ = 0.10 in the benzo[*d*][1,3]dioxole region. The corresponding Fukui isosurface shows spatially differentiated regions associated with electron density acceptance and loss, with the quinoline and benzodioxole portions displaying distinct contributions. In contrast to **EP01**, the saturated propanol chain showed little involvement in the Fukui isosurface, consistent with the interruption of π–conjugation. Thus, the local reactivity pattern of **EP02** is characterized by a greater spatial separation between electronically differentiated regions.

For **EP03**, two sites reached the threshold for electron density acceptance, N7 and C10 (*f*^+^ = 0.10), both located within the quinoline framework, whereas O19 (*f*^−^ = 0.10) was located in the benzo[d][1,3]dioxole fragment. This spatial separation is also apparent in the Fukui isosurface (Figure 2), indicating that the quinoline and benzodioxole moieties play different roles in the local electronic response. Interestingly, this organization resembles that observed for **EP02**, consistent with the common direct connection between the quinoline and benzodioxole fragments, although the substitution of the quinoline framework modifies the magnitude and distribution of the local contributions.

**EP04** exhibits a distinct local–reactivity pattern in which the largest condensed Fukui contributions are concentrated within the quinoline framework. The sites reaching the selected threshold for electron–density acceptance are N7 and C10 (*f*^+^ = 0.10), whereas the largest contributions associated with electron–density donation are located at C1 and C6 (*f*^−^ = 0.11). In contrast, none of the atoms of the 4–chlorophenyl substituent reached the 0.10 threshold for either Fukui function. This distribution indicates that, despite the direct conjugation between the quinoline and chlorophenyl rings, the dominant local response predicted by the condensed Fukui descriptors remains concentrated on the quinoline moiety. The corresponding isosurface therefore reflects an electronically differentiated quinoline framework rather than a local–reactivity pattern centered at the junction between the two aromatic fragments.

Spin–density distributions provide complementary information by indicating where the unpaired electron is accommodated after one–electron reduction or oxidation. In the radical anion of **EP01**, the largest contributions occurred at C12 (ρspin = 0.18), N7 (0.16), and C8 (0.13). These sites extended from the quinoline region toward the vinylene bridge, in agreement with the spatial distribution observed in the radical anion isosurface (Figure 3). Upon oxidation, the distribution shifted toward the opposite portion of the conjugated system, and the radical cation exhibited major contributions at C16 (0.18), C11 (0.15), C13 (0.14), O20 (0.13), and C14 (0.12). The radical cation isosurface (Figure 4) also shows a substantial spin density over the vinylene/oxygenated aromatic region. Thus, reduction and oxidation preferentially involve different regions of **EP01**, but these regions remain connected through a continuous π–conjugated framework.

For **EP02** and **EP03**, the radical anion spin density was predominantly associated with the quinoline framework. **EP02** showed major contributions at N7 (0.19), C10 (0.17), C3 (0.12), and C8 (0.10), whereas **EP03** displayed a closely related pattern at N7 (0.19), C10 (0.18), C3, and C8 (0.11). In both molecules, the radical–cation distribution shifted toward the benzo[d][1,3]dioxole–containing region. For **EP02**, the largest contributions occurred at C18 (0.17), C16 and O23 (0.14), C15 (0.13), C19 (0.11), and O21 (0.10); for **EP03**, they occurred at C14 (0.17), C19 (0.16), C11 and C12 (0.13), C15 (0.12), and O17 (0.11). The spin–density isosurfaces in Figure 3 and Figure 4 visually reproduce this redistribution, supporting a common pattern in which reduction is preferentially stabilized by the quinoline region, while oxidation shifts the unpaired–electron density toward the second aromatic fragment.

Finally, **EP04** displays a clear redistribution of spin density within the quinoline framework upon reduction and oxidation. In the radical anion, the principal contributions are located at N7 (ρspin = 0.20), C10 (0.17), and C8 (0.14), indicating preferential accommodation of the added electron around the nitrogen–containing portion of the quinoline system. In contrast, the radical cation exhibits its largest spin–density contributions at C1 (0.22), C6 (0.18), and C3 (0.16), corresponding to the carbocyclic portion of the quinoline framework. Thus, unlike **EP02** and **EP03**, the oxidation of **EP04** does not produce a pronounced shift in the unpaired–electron density toward the second aromatic substituent. Instead, both one–electron reduction and oxidation are predominantly accommodated within different regions of the quinoline moiety, while the 4–chlorophenyl group makes comparatively smaller atomic contributions. This behavior is consistent with the condensed Fukui analysis and indicates that, despite direct conjugation between the quinoline and phenyl rings, the quinoline framework remains the principal electronic region involved in the one–electron response of **EP04**.

### 2.3. Experimental and Computational Spectroscopic Analysis

The experimental UV–Vis spectra recorded in methanol show that **EP01** has the most bathochromically shifted absorption in the series, with a maximum at 353 nm (Figure 5 and Table 3). Its vinylene bridge connects the substituted aromatic ring to the quinoline unit, extending the π–conjugated pathway. This structural feature is consistent with the smallest calculated HOMO–LUMO gap in the series (5.65 eV) and an intense S_1_ transition at 383 nm (*f* = 1.57), dominated by the HOMO → LUMO excitation (89%). Although the calculated transition is red–shifted by 30 nm relative to the experimental maximum, both results identify **EP01** as the chromophore with the longest–wavelength response.

**EP02** shows the opposite behavior: its principal experimental band occurs at 265 nm, 88 nm blue–shifted relative to the maximum of **EP01**. In **EP02**, the benzodioxole fragment is directly attached to quinoline, while the propanol substituent does not extend the conjugation of the chromophore. Its larger HOMO–LUMO gap (6.46 eV) is consistent with the principal absorption at a higher energy. The most intense calculated transition occurs at 274 nm (*f* = 0.78), close to the experimental maximum. Thus, the **EP01–EP02** comparison associates the bathochromic shift primarily with the longer and more continuous conjugated pathway in **EP01**.

The other two compounds illustrate why the transition energy and band intensity must be considered separately. **EP03**, which combines quinoline and benzodioxole without the vinylene bridge present in **EP01**, has an intermediate HOMO–LUMO gap (6.01 eV) and a calculated HOMO → LUMO transition at 374 nm (*f* = 0.45). Its most intense calculated transition, however, occurs at 268 nm (*f* = 1.15), close to the experimental maximum at 260 nm; an experimental band is also observed at 338 nm. **EP04** contains a 4–chlorophenyl group directly attached to quinoline. Although its experimental band at 348 nm is almost as bathochromically shifted as that of **EP01**, it has the largest HOMO–LUMO gap in the series (6.50 eV).

The calculated low–energy transition at 356.9 nm has a modest oscillator strength (*f* = 0.36), whereas the most intense transition occurs at 308.9 nm (*f* = 1.43), close to the experimental band at 303 nm. The absorption behavior of **EP04** therefore shows that the frontier–orbital gap alone does not predict the ordering of the experimental absorption maxima; the orbitals involved in each transition and its oscillator strength must also be considered.

Table 4 shows how solvent polarity affects the spectral differences. For all four compounds, the calculated wavelength of the most intense transition decreased from toluene to water, corresponding to a hypsochromic shift. The effect was largest for **EP01** (390 → 383 nm; Δλ = 7 nm), followed by **EP04** (312 → 308 nm; 4 nm), **EP02** (277 → 274 nm; 3 nm), and **EP03** (270 → 268 nm; 2 nm). Even for **EP01**, this change is small compared to the 113 nm separating the most intense calculated transitions of **EP01** and **EP02** in toluene. Accordingly, the wavelength ordering of **EP01** > **EP04** > **EP02** > **EP03** was preserved across all solvents examined.

Within the SMD treatment, the more polar environment is associated with a slight increase in transition energy, while the spectral ordering associated with the chromophores’ structures remains unchanged. The somewhat greater solvent sensitivity of **EP01** is consistent with its extended π–conjugated pathway, through which excitation may redistribute electron density. Nevertheless, wavelengths alone cannot quantify intramolecular charge transfer or establish whether the shift arises from preferential stabilization of the ground or excited state. Table 4 also reports the most intense transition, which is not necessarily S_1_. This distinction is particularly relevant to **EP03** and **EP04**, whose lower–energy transitions occur at longer wavelengths than those listed in that table.

### 2.4. Nonlinear Optical Properties

The calculated NLO parameters revealed substantial differences across the **EP01**–**EP04** series and relative to previously reported quinoline derivatives (Table 5). Among the studied compounds, **EP01** exhibited the highest average polarizability (α_ave_ = 63.10 × 10^−24^ esu), first hyperpolarizability (β_tot_ = 118.95 × 10^−30^ esu), and second hyperpolarizability (γ_ave_ = 498.55 × 10^−36^ esu). **EP02**, **EP03**, and **EP04** showed progressively lower β_tot_ values of 46.02, 15.74, and 5.60 × 10^−30^ esu, respectively, although the remaining NLO descriptors did not follow the same ordering. In particular, **EP04** combined the largest dipole moment of the series (5.47 D) with the lowest β_tot_ (5.60 × 10^−30^ esu), the lowest average polarizability (48.65 × 10^−24^ esu), and the lowest second hyperpolarizability (154.89 × 10^−36^ esu). Its polarizability anisotropy (Δα = 41.06 × 10^−24^ esu) was intermediate within the series. These results demonstrated that permanent polarity, linear polarizability, anisotropy, and higher–order NLO responses did not vary proportionally across this structural series.

Comparison with previously reported quinoline derivatives provides additional structural context. The 3–arylated quinoline reported by Khalid et al. and included in Table 5 exhibits β_tot_ = 25.57 × 10^−30^ esu [3], while the nitro–substituted styrylquinoline reported by Noudem et al. reaches β_tot_ = 61.32 × 10^−30^ esu and γ = 301.89 × 10^−36^ esu [5]. Structurally, the Khalid system contains a quinoline directly connected to a substituted phenyl ring, whereas the Noudem et al. compound incorporates a styryl bridge and a strongly electron–withdrawing NO_2_ group. **EP01** also contains a styrylquinoline framework but bears methoxy and ethoxy substituents on the opposite aromatic fragment. Within the limitations imposed by the different computational protocols, **EP01** therefore shows a notably larger calculated β_tot_ than both reference systems and a larger γ than the Noudem styrylquinoline. **EP02** also exceeds the β_tot_ reported for the Khalid derivative but remains below the Noudem system, whereas **EP03** and **EP04** show β_tot_ values closer to that of the directly linked arylquinoline.

These comparisons suggest a relationship between molecular architecture and the calculated NLO response without requiring attribution to a single structural feature. **EP01** combines the longest conjugated pathway of the present series with alkoxy substitution and exhibits the largest β_tot_ and γ. The importance of the styryl framework is also supported qualitatively by the Noudem reference, for which the introduction of an NO_2_ substituent into a styrylquinoline scaffold was associated with β_tot_ = 61.32 × 10^−30^ esu and γ = 301.89 × 10^−36^ esu. Conversely, **EP03** and **EP04**, which lack the vinylene spacer of **EP01**, exhibit much smaller β_tot_ values despite **EP04** having a large dipole moment and Δα. Thus, the present data are consistent with NLO behavior being influenced by the combined effects of π–conjugation, substituent identity, and molecular connectivity rather than by dipole moment alone.

The MEP maps reveal distinct electrostatic patterns across the **EP01**–**EP04** series (Figure 6), reflecting the differences in the substitution and molecular connectivity. **EP01** displays a pronounced spatial separation of electrostatic regions across its extended styrylquinoline framework, whereas this distribution is less continuous in **EP02** and **EP03**, where the benzodioxole–containing fragments adopt a less–extended molecular arrangement. **EP04**, despite its simpler structure, exhibits marked electrostatic differentiation across the conjugated quinoline–chlorophenyl framework. Notably, these patterns do not parallel the calculated dipole moments or NLO parameters: **EP04** exhibits the largest μ (5.47 D), whereas **EP01** exhibits the highest β_tot_ and γ_ave_. Thus, the MEP analysis reinforces that the magnitude of the NLO response cannot be rationalized solely in terms of static charge separation but must be considered together with molecular connectivity and the electronic response descriptors discussed above.

Finally, a comparison with the electronic–structure descriptors provides further insight into the NLO behavior of the series. The HOMO–LUMO gaps increase in the order **EP01** (5.65 eV) < **EP03** (6.01 eV) < **EP02** (6.46 eV) < **EP04** (6.50 eV), whereas the first hyperpolarizabilities decrease in the order **EP01** (118.95 × 10^−30^ esu) > **EP02** (46.02 × 10^−30^ esu) > **EP03** (15.74 × 10^−30^ esu) > **EP04** (5.60 × 10^−30^ esu). Thus, **EP01** combines the smallest frontier–orbital gap with the largest βtot, whereas **EP04** combines the largest gap with the smallest β_tot_. Nevertheless, the behavior of the two intermediate compounds shows that the relationship is not simply monotonic: **EP03** has a smaller HOMO–LUMO gap than **EP02** but a substantially lower βtot. Consequently, frontier–orbital separation contributes to the electronic response but does not by itself determine the magnitude of the nonlinear optical properties.

Therefore, the distinctive behavior of **EP01** should be considered in the context of its overall electronic architecture. In addition to its smaller frontier orbital gap, the HOMO and LUMO are distributed over an extended π–framework comprising the substituted aromatic ring, vinylene spacer, and quinoline moiety. In **EP02** and **EP03**, the frontier orbitals also involve the quinoline and benzodioxole fragments, but their distribution is less extended over the complete molecular framework, whereas **EP04** presents delocalization over the directly connected quinoline–phenyl system. Accordingly, the exceptionally large β_tot_ of **EP01** cannot be attributed to a single electronic descriptor but coincides with the combination of a relatively small frontier orbital gap, extended π–conjugation, and a molecular connectivity that electronically links the substituted aromatic and quinoline regions. Therefore, the comparison among **EP01**–**EP04** indicates that both orbital energetics and the spatial organization of the frontier orbitals must be considered when rationalizing the calculated NLO responses.

## 3. Experimental

### 3.1. Materials and Methods

All chemicals used in the analysis were of analytical grade and purchased from Merck and Sigma–Aldrich, Germany. ^13^C, ^1^H, and DEPT–135 NMR spectra and 2D COSY, HSQC, and HMBC experiments were obtained using a 400 MHz Bruker spectrometer (400 MHz for ^1^H and 75 MHz for ^13^C) with deuterated chloroform (CDCl_3_) as the solvent and tetramethylsilane [(CH_3_)_4_Si] as the internal standard. Chemical shifts (δ) are expressed in parts per million (ppm), and the coupling constants (*J*) are expressed in hertz (Hz). IR spectra were recorded using a Nicolet FTIR spectrometer with diamond ATR. Silica gel 60 (0.063–0.200 mm) was used for column chromatography, and pre–coated silica gel plates (Merck 60 F254 0.2 mm) were used for thin–layer chromatography (TLC) to monitor the reaction progress.

### 3.2. Synthesis

#### 3.2.1. (E)–2–(4–ethoxy–3–methoxystyryl)quinoline (**EP01**)

For the chemical synthesis, a 1:1 stoichiometric mixture of quinaldine (2–methylquinoline, 98%, 1.3541 mL, 10 mmol) and the corresponding aromatic aldehyde (4–ethoxy–3–methoxybenzaldehyde, 98%, 1.8064 g, 10 mmol) was dissolved in acetic anhydride [21]. The resulting solution was heated at reflux for 24 h. After completion of the reaction, the mixture was allowed to cool to room temperature and carefully neutralized with sodium bicarbonate. The product was extracted using ethyl acetate. The combined organic layers were dried over anhydrous sodium sulfate, filtered, and concentrated under reduced pressure to obtain a crude product. Purification was carried out by column chromatography (CC) using a gradient elution of n–hexane/ethyl acetate (increasing polarity), yielding 0.623 g of the desired product. Brown crystals; yield: 21%; m. p. 121–123 °C; ^1^H NMR (CDCl_3_, 400 MHz): 1.49 (t, *J* = 8.5 Hz, 3H), 3.94 (s, 3H) 4.14 (q, *J* = 7.0 Hz, 2H), 6.88 (d, *J* = 8.6 Hz, 1H), 7.13 (dd, *J* = 1.9, 8.2 Hz, 1H), 7.23 (d, *J* = 1.9 Hz, 1H), 7.29 (d, *J* = 16.4 Hz, 1H), 7.48 (t, *J* = 7.0 Hz, 1H), 7.59 (d, *J* = 16.4 Hz, 1H), 7.66–7.71 (m, 2H), 7.76 (d, *J* = 8.2 Hz, 1H), 8.08 (dd, *J* = 8.6, 15.6 Hz, 1H), ^13^C– NMR (CDCl_3_): (ppm) 14.7 (CH_3_), 55.8 (OCH_3_), 64.2 (CH_2_), 108.9 (CH), 112.2 (CH), 118.7 (CH), 121.2 (CH), 125.9 (CH), 126.9 (CH), 127.1 (C), 127.4 (CH), 128.9 (CH), 129.3 (C), 129.6 (CH), 134.2 (CH), 136.2 (CH), 148.1 (C), 149.0 (C), 149.3 (C), 156.2 (C).

#### 3.2.2. c 3–(2–(Benzo[d][1,3]dioxol–5–yl)–6–fluoroquinolin–3–yl)propan–1–ol (**EP02**)

Initially, 1 mmol of 4–fluoroaniline, 1.2 mmol of piperonal, and 10 mL of ethanol (EtOH) were added to a 25 mL round–bottomed flask. The reaction mixture was heated under reflux at approximately 70 °C for 2 h. The formation of the imine intermediate was monitored using thin–layer chromatography (TLC). After completion of the reaction, the mixture was allowed to cool to room temperature for 10 min, and the solvent was removed under reduced pressure. The resulting crude residue was dissolved in 10 mL of acetonitrile (MeCN) without further purification. Subsequently, 3 mmol of 3,4–dihydro–2*H*–pyran and BiCl_3_ (20 mol%) was added. The reaction mixture was stirred at room temperature for 18 h, and the formation of the tetrahydroquinoline (THQ) intermediate was confirmed using TLC.

Finally, 4 mmol of manganese dioxide (MnO_2_) was added directly to the reaction mixture, which was heated under reflux for an additional 2 h. Upon completion of the reaction, the formation of the corresponding quinoline derivative was confirmed by TLC.

Brown crystals; yield: 53%; m. p. 135–136 °C; ^1^H NMR (CDCl_3_, 400 MHz): 1.62–1.61 (m, 2H), 2.78–2.82 (m, 2H), 3.32–3.36 (m, 2H), 4.48 (t, *J* = 4.9 Hz, 1H), 6.10 (s, 2H), 7.01 (s, 2H), 7.11 (s, 1H), 7.56–7.61 (m, 1H), 7.72 (dd, *J* = 2.5, 9.6 Hz, 1H), 8.00 (dd, *J* = 5.5, 9.4 Hz, 1H), 8.22 (s, 1H); ^13^C– NMR (CDCl_3_): 28.9 (CH_2_), 32.7 (CH_2_), 60.0 (CH_2_), 101.2 (CH_2_), 107.9 (CH), 109.3 (CH), 110.1 (CH, 21.1), 118.8 (CH, *J*_C–F_ = 26.2 Hz), 122.6 (CH), 127.7 (C, *J*_C–F_ = 10.2 Hz), 131.3 (CH, *J*_C–F_ = 9.4 Hz), 134.2 (C), 134.4 (C), 135.0 (CH, 5.1), 142.9 (C), 147.0 (C, 12.4), 158.4 (C), 158.9 (C, *J*_C–F_ = 2.9 Hz), 160.9 (C).

#### 3.2.3. Synthesis of 2–(Benzo[d][1,3]dioxol–5–yl)–6–methoxyquinoline (**EP03**)

A solution of 4–methoxyaniline (2.8 mmol) and piperonal (3.4 mmol) in anhydrous MeCN (15 mL) was stirred at room temp. for 30 min and BiCl_3_ (20 mol%) was added. Over a period of 20 min, a solution of *N*–vinyl–2–pyrrolidone (NVP) (5.5 mmol) in MeCN (10 mL) was added dropwise. The resulting mixture was then stirred for 10–14 h. After completion of the reaction, as indicated by TLC, the reaction mixture was diluted with water (30 mL) and extracted with ethyl acetate (EtOAc) (3 × 15 mL). The organic layer was separated and dried (Na_2_SO_4_), concentrated under vacuum, to afford the respective 2–aryl–1,2,3,4–tetrahydroquinolines, which were used in subsequent synthetic steps without careful purification. The corresponding 2–aryl–1,2,3,4–tetrahydroquinolines were heated quickly (10–15 min) in the presence of elemental sulfur S8 to 220–230 °C, and the reaction mixture was adsorbed on silica gel and separated by column chromatography to afford the 2–(benzo[*d*][1,3]dioxol–5–yl)–6–methoxyquinoline (**EP03**) [22,23].

Brown crystals; yield: 59%; m. p. 139–141 °C; ^1^H NMR (CDCl_3_, 400 MHz); 3.93 (s, 3H), 6.02 (s, 2H), 6.93 (d, *J* = 8.2 Hz, 1H), 7.06 (d, *J* = 2.7 Hz, 1H), 7.36 (dd, *J* = 2.7, 9.4 Hz, 1H), 7.60 (dd, *J* = 1.6, 8.2 Hz, 1H), 7.69 (d, *J* = 1.6 Hz, 1H), 7.74 (d, *J* = 8.6 Hz, 1H), 8.01 (d, *J* = 9.4 Hz, 1H), 8.06 (d, *J* = 8.6 Hz, 1H); ^13^C– NMR (CDCl_3_): 55.5 (OCH_3_), 101.2 (CH_2_), 104.9 (CH), 107.6 (CH), 108.4 (CH), 118.8 (CH), 121.3 (CH), 122.2 (CH), 127.8 (C), 130.9 (CH), 134.2 (C), 135.4 (CH), 144.1 (C), 148.3 (C), 148.4 (C), 154.4 (C), 157.4 (C).

#### 3.2.4. Synthesis of 2–(4–Chlorophenyl)–6–methylquinoline (**EP04)**

A solution of 4–metylaniline (2.8 mmol) and 4–chlorobenzaldehyde (3.4 mmol) in anhydrous MeCN (15 mL) was stirred at room temp. for 30 min and BiCl_3_ (20 mol%) was added. Over a period of 20 min, a solution of *N*–vinyl–2–pyrrolidone (NVP) (5.5 mmol) in MeCN (10 mL) was added dropwise. The resulting mixture was stirred for 10–14 h. After completion of the reaction, as indicated by TLC, the reaction mixture was diluted with water (30 mL) and extracted with ethyl acetate (EtOAc) (3 × 15 mL). The organic layer was separated and dried (Na_2_SO_4_), concentrated under vacuum, to afford the respective 2–aryl–1,2,3,4–tetrahydroquinolines, which were used in subsequent synthetic steps without careful purification. The corresponding 2–aryl–1,2,3,4–tetrahydroquinolines were heated quickly (10–15 min) in the presence of elemental sulfur S8 to 220–230 C, and the reaction mixture was adsorbed on silica gel and separated by column chromatography to afford the 2–(4–chlorophenyl)–6–methylquinoline (**EP04**) [24].

Green crystals; yield: 51%; m. p. 164–166 °C [24,25], ^1^H NMR (CDCl_3_, 400 MHz): 2.55 (s, 3H), 7.48 (d, *J* = 8.6 Hz, 2H), 7.56–7.59 (m, 1H), 7.79 (d, *J* = 8.6 Hz, 1H), 8.04 (d, *J* = 8.2 Hz, 1H), 8.09–8.14 (m, 3H); ^13^C– NMR (CDCl_3_): 21.6 (CH_3_), 118.5 (CH), 126.3 (CH), 127.2 (C), 128.6 (2xCH), 128.9 (2xCH), 129.3 (CH), 132.1 (CH), 135.2 (C), 136.2 (CH), 136.4 (C), 138.1 (C), 146.7 (C), 155.1 (C).

### 3.3. Single–Crystal X–Ray Diffraction Analysis

Crystals of **EP01**, **EP02**, **EP03**, and **EP04** suitable for X–ray analysis were obtained as previously reported. We selected a morphologically well–defined specimen and affixed it to a MicroMount in a non–specific orientation. X–ray intensity data were acquired at 296 K on a Bruker D8 VENTURE diffractometer fitted with a PHOTON III C14 CMOS area detector using IµS 3.0 microfocus Mo–Ka radiation (l = 0.71073 Å). Raw diffraction frames were collected and processed using the Bruker APEX software suite and integrated with SAINT V8.40B; empirical absorption corrections were applied using SADABS–2016/2 [26]. The crystal structures in all four cases were solved using the Intrinsic Phasing approach implemented in ShelXT [27] and subsequently refined against *F*^2^ by full–matrix least–squares methods using ShelXL [27]. All structure solution and refinement procedures were performed using the Olex2 1.5 graphical interface [28]. All non–hydrogen atoms were refined with anisotropic displacement parameters. With the exception of the hydroxyl hydrogen atom in **EP02** (H3), which was located from the difference Fourier map and refined isotropically with the OH distance restrained to a target value, all remaining hydrogen atoms in the four structures were placed in geometrically calculated positions and refined using a riding model with isotropic displacement parameters; Uiso values for CH and CH_2_ hydrogen atoms were constrained to 1.2× the equivalent isotropic displacement parameter of the parent atom, while those of CH_3_ groups were set to 1.5×. Crystal data, data collection statistics, and refinement parameters for the four structures are compiled in Table S9; the complete crystallographic datasets are deposited as CIF files with the CCDC numbers 2561078 (**EP01**), 2573280 (**EP02**), 2586271 (**EP03**), and 2586270 (**EP04**). Molecular graphics were produced using DIAMOND, and geometric calculations of intermolecular distances and angles were performed using Mercury [28,29].

### 3.4. Computational Studies

#### DFT Calculations

All molecular geometries were optimized using the ORCA 6.1.1 software package [30]. The optimized geometries corresponded to the true minima on the potential energy surface, as verified by the absence of imaginary frequencies in the harmonic vibrational analysis [31]. Geometry optimization and ground–state electronic property calculations were performed using the M06–2X hybrid meta–GGA functional with a def2–TZVPP basis set. The solvent effects were included using the SMD implicit solvation model with methanol as the solvent model. M06–2X, which incorporates 54% Hartree–Fock exchange, was selected for its reliable performance in molecular geometries, thermochemistry, and noncovalent interactions [32].

The electron affinity (EA) and ionization potential (IP) were calculated using the adiabatic method [31,33,34]. In the adiabatic approach, the geometries of the charged species (cations and anions) were fully optimized prior to the energy calculations, thus accounting for structural relaxation upon electron detachment or attachment. The corresponding expressions are as follows:

*EA* = *G_Neutral_* − *G_anion_*

*IP* = *G_Cation_* − *G_Neutral_*

where *G_neutral_, G_anion_,* and *G_cation_* are the Gibbs free energies of the corresponding fully optimized species.

Chemical hardness was calculated according to the definition proposed by Parr and Pearson, which involves differentiating the chemical potential with respect to the number of electrons at a constant energy potential. The latter can be approximated as half the difference between the ionization potential (IP) and electron affinity (EA) [35]. To achieve symmetry and ensure electronegativity symmetry, the product was multiplied by half, as highlighted by Pearson [36].

$$\eta =(\frac{\partial^{2}E}{\partial^{2}N}{)}_{v(r)}\approx \frac{\mathrm{IP}-\mathrm{EA}}{2}$$

The electrophilicity index was calculated as follows:

$$\omega =\frac{x^{2}}{2\eta }$$

where

$$x=-(\frac{\partial E}{\partial N}{)}_{v(r)}\approx \frac{\mathrm{IP}+\mathrm{EA}}{2}$$

The condensed nucleophilic Fukui function $f_{k}^{+}$ identifies the atoms in a molecule that are more susceptible to nucleophilic attack. It was calculated using the finite difference approximation as follows:

$$f_{k}^{+}=\mathrm{qk}\left(N\right)-\;\mathrm{qk}\left(N+1\right)$$

where *qk*(*N*) and *qk*(*N* + 1) are the Hirshfeld charges of atom *k* in neutral and anionic species, respectively [37,38].

The condensed electrophilic attack Fukui function $f_{k}^{-}$ identifies atoms in a molecule that are more susceptible to electrophilic attack. This was calculated using the finite difference approximation as follows:

$$f_{k}^{-}=\mathrm{qk}\left(N-1\right)-\mathrm{qk}(N)$$

where *qk*(*N*) and *qk*(*N* − 1) are the Hirshfeld charges of atom *k* in neutral and cationic species, respectively [37,38].

The condensed spin density $\rho_{k}^{\mathrm{spin}}\;$ is defined as the difference between the alpha and beta electron densities of each atom *k* in the radical anion or cation:

$$\rho_{k}^{\mathrm{spin}}=\rho_{k}^{\alpha }\;-\;\rho_{k}^{\beta }$$

This descriptor provides insight into the delocalization of unpaired electrons and the radical stabilization potential [38]. All condensed values were obtained using Hirshfeld population analysis, as implemented in ORCA [39].

Electronic transitions to the first 50 excited states were computed using TD–DFT at the CAM–B3LYP/6–311++G(d,p) level, with solvent effects incorporated through the SMD implicit solvation model for toluene, dichloromethane (DCM), 2–propanol (i–PrOH), ethanol (EtOH), methanol (MeOH), and water [31,40].

The nonlinear optical (NLO) response parameters were evaluated using Gaussian 16 at the CAM–B3LYP/6–311++G(d,p) level of theory. CAM–B3LYP was selected because its range–separated treatment of exchange is suitable for describing the electronic polarization and charge redistribution relevant to the NLO properties. The solvent effects were included using the SMD implicit solvation model with methanol as the solvent.

Consequently, using the finite field approach and as widely reported in the literature, parameters such as the average dipole moment (*µ*), linear (α_0_), total (Δα) polarizability, and first–order (β*_tot_*) and second–order (γ*_ave_*) hyperpolarizabilities were calculated according to the following equations [9].

$$\mu ={\left(\mu_{x}^{2}+\mu_{y}^{2}+\mu_{z}^{2}\right)}^{1/2}$$

Linear polarizability

$$\alpha_{0}=\frac{1}{3}(\alpha_{\mathrm{xx}}+\alpha_{\mathrm{yy}}+\alpha_{\mathrm{zz}})$$

Polarizability anisotropy

$$∆\alpha =\frac{1}{\sqrt{2}}\sqrt{{\left(\alpha_{\mathrm{xx}}-\alpha_{\mathrm{yy}}\right)}^{2}+{\left(\alpha_{\mathrm{yy}}-\alpha_{\mathrm{zz}}\right)}^{2}+{\left(\alpha_{\mathrm{zz}}-\alpha_{\mathrm{xx}}\right)}^{2}+6({\alpha_{\mathrm{xy}}}^{2}+{\alpha_{\mathrm{yz}}}^{2}+{\alpha_{\mathrm{xz}}}^{2})}$$

First–order hyperpolarizability

$$\beta_{\mathrm{tot}}={\left[{\left(\beta_{\mathrm{xxx}}+\beta_{\mathrm{xyy}}+\beta_{\mathrm{xzz}}\right)}^{2}+{\left(\beta_{\mathrm{yyy}}+\beta_{\mathrm{yzz}}+\beta_{\mathrm{yxx}}\right)}^{2}+{\left(\beta_{\mathrm{zzz}}+\beta_{\mathrm{zxx}}+\beta_{\mathrm{zyy}}\right)}^{2}\right]}^{1/2}$$

Second–order hyperpolarizability

$$\gamma_{\mathrm{ave}}=\frac{1}{5}\left[\gamma_{\mathrm{xxxx}}+\gamma_{\mathrm{yyyy}}+\gamma_{\mathrm{zzzz}}+2\left[\gamma_{\mathrm{xxyy}}+\gamma_{\mathrm{yyzz}}+\gamma_{\mathrm{xxzz}}\right]\right]$$

## 4. Conclusions

A comparison of **EP01**–**EP04** shows that the electronic and optical responses of these quinoline derivatives depend on π–system connectivity and substituent identity**. EP01**, whose vinylene bridge connects the substituted aromatic ring to the quinoline unit, exhibited the smallest HOMO–LUMO gap in the series (5.65 eV) and the most bathochromically shifted experimental absorption in methanol (353 nm). The calculated S_1_ transition was also the most intense among the low–energy transitions examined (383 nm; *f* = 1.57). Compared with **EP02**, whose principal absorption maximum occurred at 265 nm, these findings are consistent with more extensive electronic communication across the **EP01** chromophore.

However, a comparison with **EP03** and **EP04** shows that the HOMO–LUMO gap alone does not account for the position or intensity of every absorption band**. EP04** displayed a band at 348 nm despite having the largest calculated gap (6.50 eV), whereas the most intense transitions of **EP03** and **EP04** were not their lowest–energy transitions. Calculations across different solvents predicted small hypsochromic shifts of 2–7 nm for the most intense transitions from toluene to water in the UV–Vis spectra. Thus, the differences among the chromophores were more pronounced than the calculated effect of the solvent polarity.

This relationship between structure and electronic response was also reflected in the calculated nonlinear optical properties**. EP01** had the largest first hyperpolarizability in the series (βtot = 118.95 × 10^−30^ esu), approximately 2.58 times that of **EP02**, as well as the largest second hyperpolarizability (γave = 498.55 × 10^−36^ esu). By contrast, **EP04** combined the largest dipole moment with the lowest βtot, indicating that permanent polarity alone does not predict the nonlinear response. Taken together, these findings identify the vinylene bridge and the electronic integration of the aromatic fragments in **EP01** as promising structural features for designing new quinoline chromophores. Experimental evaluation of their nonlinear optical properties is needed to establish their performance as materials.

## Figures and Tables

**Figure 1:**
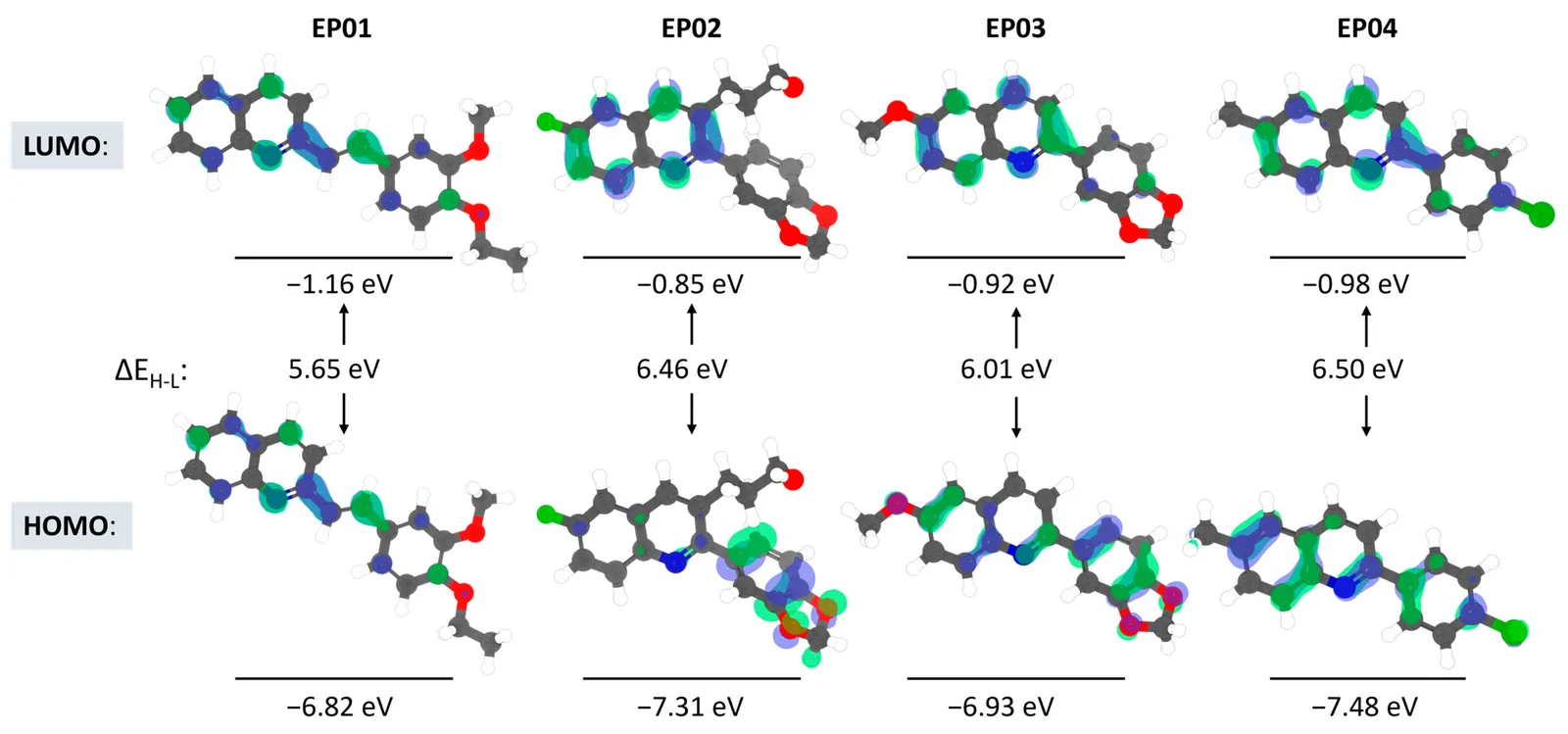
HOMO and LUMO frontier molecular orbitals (eV) of EP01–EP04. Calculated at the M06–2X/def2–TZVPP level using the SMD implicit solvation model in methanol. The isosurface was calculated using a value of 0.03 a.u. The blue and green colors represent the positive and negative phases of the molecular orbital wavefunctions (HOMO and LUMO), respectively.

**Figure 2:**
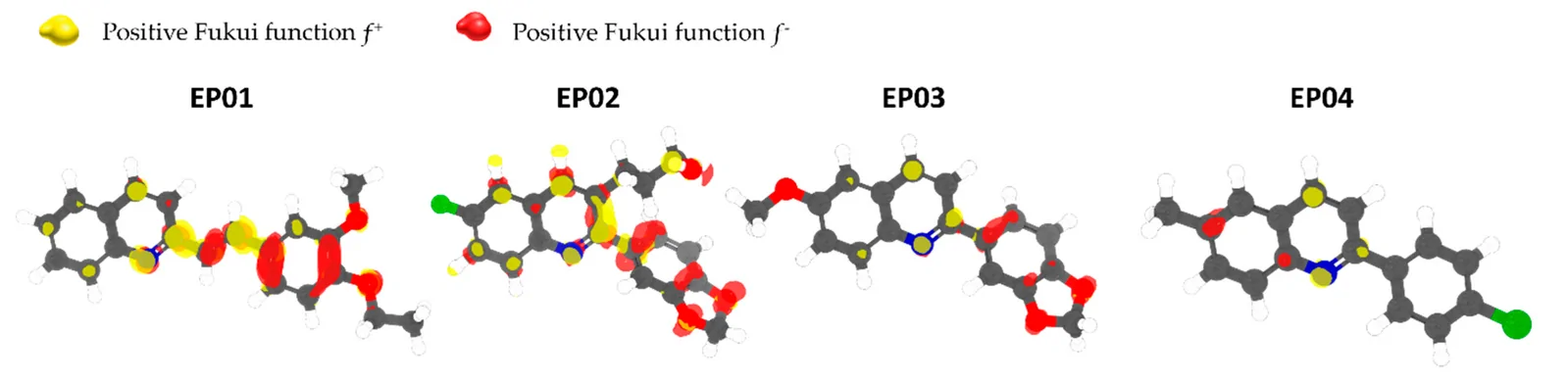
Isosurfaces of the Fukui dual descriptors of **EP01**–**EP04**. The red regions correspond to the electrophilic Fukui function (*f*^−^), and the yellow areas correspond to the nucleophilic Fukui function (*f*^+^). The isosurface of the Fukui dual descriptor was calculated at a value of 0.0025 a.u. Calculated at the M06–2X/def2–TZVPP level using the SMD implicit solvation model in methanol.

**Figure 3:**
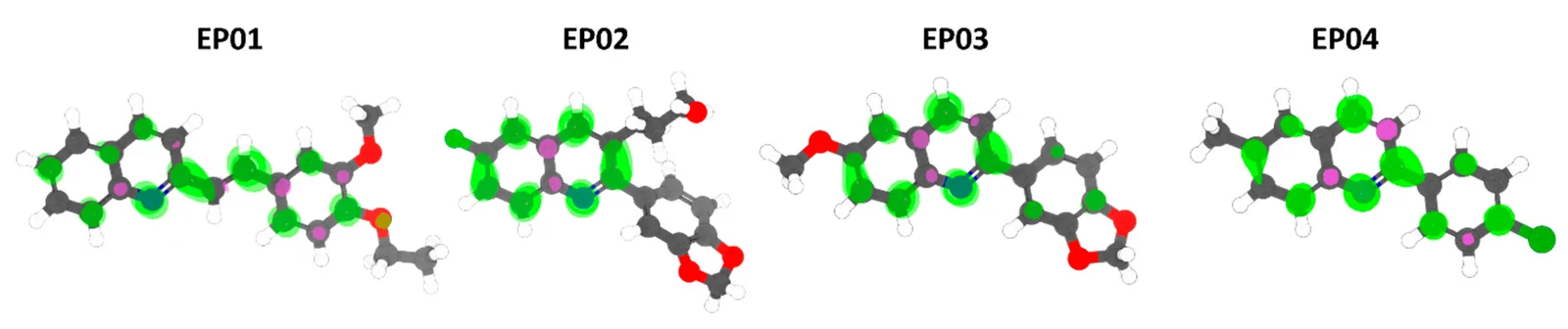
Spin density isosurfaces of the radical anion forms of **EP01**–**EP04**. The green and purple regions represent the spatial distributions of the α– and β–spin densities, respectively, illustrating the localization of the unpaired electron after single–electron reduction. The isosurfaces for the spin density were calculated using a value of 0.0035 a.u. Calculated at the M06–2X/def2–TZVPP level using the SMD implicit solvation model in methanol.

**Figure 4:**
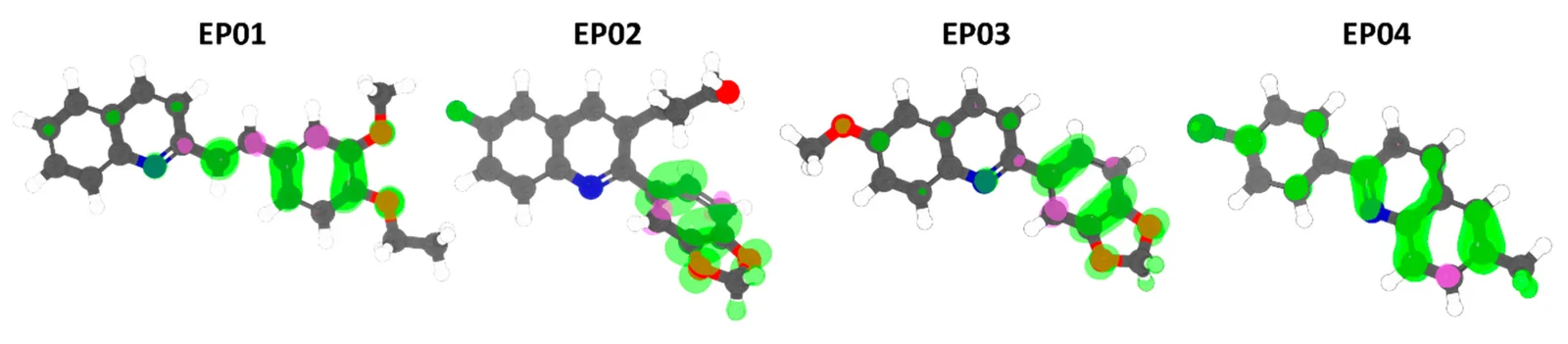
Spin density isosurfaces of the radical cation forms of **EP01**–**EP04**. The green and purple regions represent the spatial distributions of the α– and β–spin densities, respectively, illustrating the localization of the unpaired electron after the single–electron oxidation. The isosurfaces for the spin density were calculated using a value of 0.0035 a.u. Calculated at the M06–2X/def2–TZVPP level using the SMD implicit solvation model in methanol.

**Figure 5:**
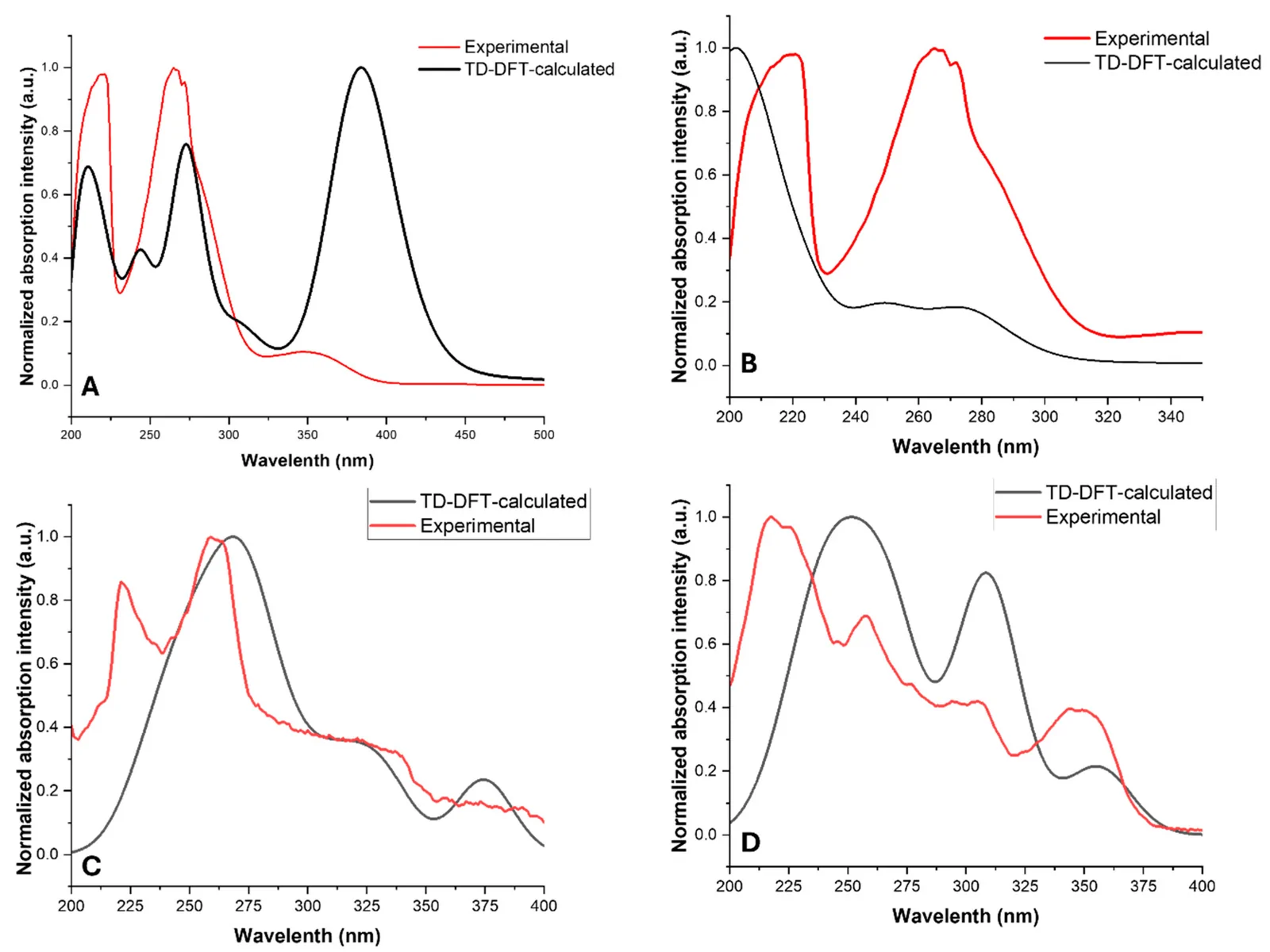
Experimental and calculated UV–Vis spectra of (**A**) **EP01**, (**B**) **EP02**, (**C**) **EP03**, and (**D**) **EP04**. The experimental UV–Vis spectra were recorded in methanol, whereas the calculated spectra were obtained using TD–CAM–B3LYP/6–311++G(d,p) with the SMD implicit solvation model in methanol.

**Figure 6:**
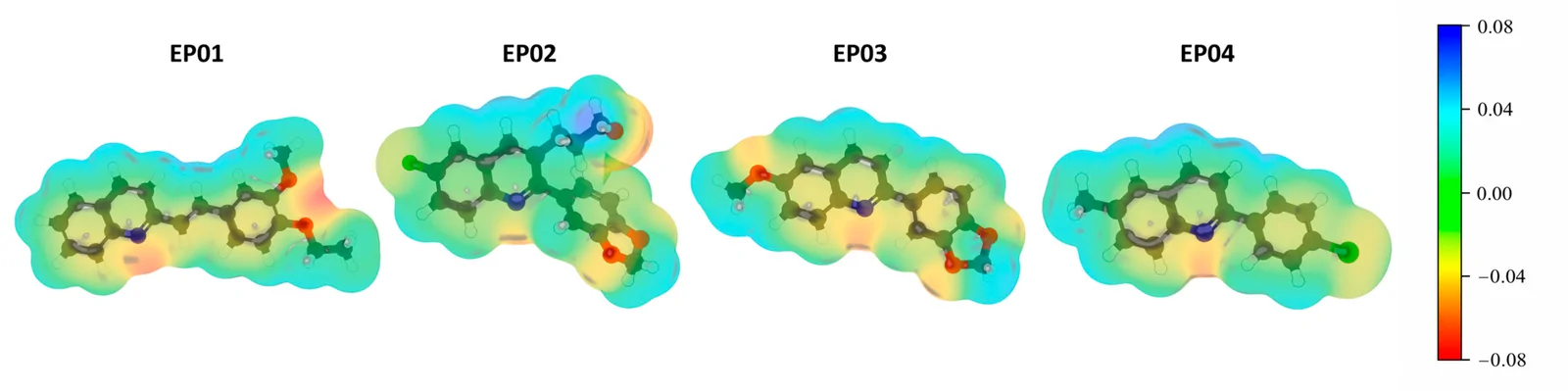
Molecular electrostatic potential (MEP) maps of **EP01–04**, calculated at the M06–2X/def2–TZVPP level using the SMD implicit solvation model in methanol. The isosurfaces were calculated using a value of 0.001 a.u. The color scale ranges from red (negative electrostatic potential, electron-rich regions) to blue (positive electrostatic potential, electron-deficient regions), with green indicating regions of approximately neutral electrostatic potential.

**Table 1:** Synthesized Quinoline Analogs.

Reaction Scheme and Conditions	X–Ray Crystal Structure (CCDC)
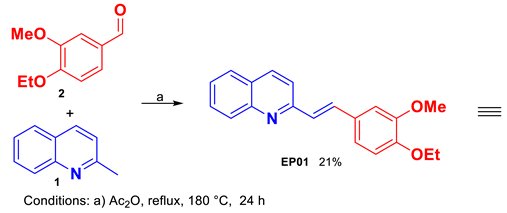	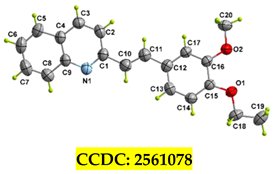
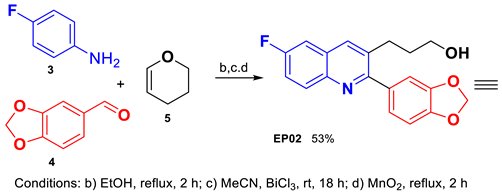	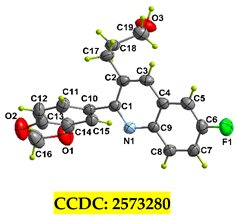
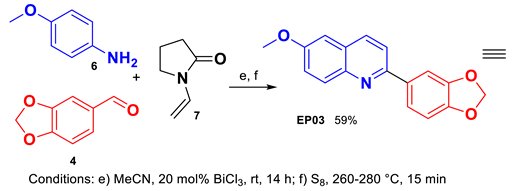	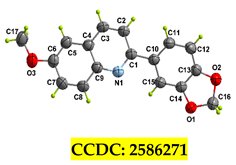
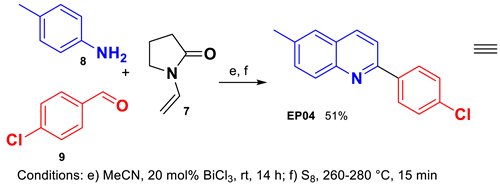	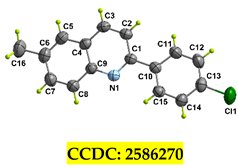

**Table 2:** Global electronic properties (eV) calculated at the M06–2X/def2–TZVPP level of theory using the SMD implicit solvation model in methanol. The table includes the energies of the highest occupied molecular orbital (HOMO, H) and the lowest unoccupied molecular orbital (LUMO, L), HOMO–LUMO energy gap (ΔE_H–L_), electron affinity (EA), ionization potential (IP), chemical hardness (η), and electrophilicity index (ω) of the corresponding species.

Molecule	HOMO	LUMO	Energy Gap (ΔE_H–L_)	IP	EA	η	ω
**EP01**	−6.82	−1.16	5.65	5.50	2.52	1.49	5.38
**EP02**	−7.31	−0.85	6.46	5.75	2.33	1.71	4.76
**EP03**	−6.93	−0.92	6.01	5.69	2.31	1.69	4.75
**EP04**	−7.48	−0.98	6.50	6.18	2.55	1.82	5.25

**Table 3:** Main electronic transitions of **EP01–04** calculated in methanol using the SMD implicit solvation model. ^a^ Calculated transition energy. ^b^ Oscillator strength. ^c^ Experimental absorption maximum measured in methanol. H: HOMO, L: LUMO.

Excited State	Excitation Vertical (nm)	E_calc_ ^a^ (eV)	*f_os_* ^b^	Major Contribution (%)	λ_abs_ ^c^ (nm)
**EP01 CAM–B3LYP/6–311++G(d,p)**					
S_1_	383	3.24	1.57	H+0 → L+0 (89%)	353
S_8_	272	4.56	1.11	H–1 → L+1 (27%)	265
S_15_	263	4.71	0.32	H+0 → L+10 (34%)	265
**EP02 CAM–B3LYP/6–311++G(d,p)**					
S_8_	274	4.52	0.78	H–2 → L+0 (22%)	265
S_17_	252	4.92	0.60	H–2 → L+1 (33%)	265
S_25_	241	5.14	0.13	H–3 → L+1 (25%)	220
**EP03 CAM–B3LYP/6–311++G(d,p)**					
S_12_	268	4.63	1.15	H–3 → L+0 (26%)	260
S_1_	374	3.32	0.45	H+0 → L+0 (83%)	–––
S_5_	320	3.87	0.43	H+0 → L+3 (24%)	338
**EP04 CAM–B3LYP/6–311++G(d,p)**					
S_4_	308	4.01	1.43	H+0 → L+1 (50.0%)	303
S_10_	266	4.65	0.87	H–1 → L+1 (24.0%)	218
S_1_	356	3.47	0.36	H+0 → L+0 (73.1%)	348

**Note**: Bold text indicates the specific compound and functional/basis set used for the calculations.

**Table 4:** Calculated wavelengths (nm) of the main (most intense) electronic transition of **EP01**–**EP04** in various solvents at the TD–CAM–B3LYP/6–311++G(d,p) level, with solvent effects described using the SMD implicit solvation model.

Compound	Toluene	DCM	i–PrOH	EtOH	MeOH	Water
**EP01**	390	389	386	385	383	383
**EP02**	277	276	275	275	274	274
**EP03**	270	269	269	268	268	268
**EP04**	312	312	310	309	309	308

**Table 5:** Calculated dipole moment (μ), polarizability (α), first–order hyperpolarizability (β_tot_), and second–order hyperpolarizability (γ_ave_) of **EP01**–**EP04**, obtained at the CAM–B3LYP/6–311++G(d,p) level using the SMD implicit solvation model in methanol.

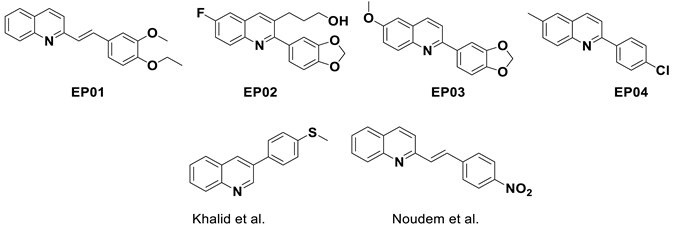					
Compound	*μ_tot_* (*D*)	*α_ave_* (×10^−24^ esu)	∆*α* (×10^−24^ esu)	*β* (×10^−30^ esu)	*γ* (×10^−36^ esu)
**EP01**	3.09	63.1	58.41	118.95	498.55
**EP02**	3.95	55.63	36.88	46.02	170.72
**EP03**	1.35	52.78	38.29	15.74	218.07
**EP04**	5.47	48.65	41.06	5.60	154.89
Khalid et al. [3]	3.29	36.30	–––	25.57	5.70
Noudem et al. [5]	6.88	39.14	56.44	61.32	301.89

## Data Availability

The original contributions presented in this study are included in the article. Further inquiries can be directed to the corresponding author.

## Supplementary Materials

The following supporting information can be downloaded at https://www.mdpi.com/article/10.3390/molecules31203753/s1. Figure S1: ^1^H–NMR compound **EP01**; Figure S2: ^13^C–NMR compound **EP01**; Figure S3: Dept–135 compound **EP01**; Figure S4: ^1^H–NMR compound **EP02**; Figure S5: ^13^C–NMR compound **EP02**; Figure S6: Dept 135 compound **EP02**; Figure S7: ^1^H–NMR compound **EP03**; Figure S8: ^13^C–NMR compound **EP03**; Figure S9: Dept 135 compound **EP03**; Figure S10: View of the asymmetric unit of **EP01** with the numbering scheme of molecular structure, where atomic displacement ellipsoids are drawn at the 50% probability level; Figure S11: Perspective view of **EP01** show the p···p interaction growing along the b–axis (Symmetry code: (i) x, y−1, z) with interactions shown as yellow dashed lines; Figure S12: View of the asymmetric unit of **EP02** with the numbering scheme of molecular structure, where atomic displacement ellipsoids are drawn at the 50% probability level; Figure S13: Perspective view of **EP02** show the hydrogen bonding interaction growing along the [1,0,1] direction (Symmetry operation: (ii) x + 1/2, −y + 3/2, z−1/2) with interactions shown as dashed lines; Figure S14: View of ac–plane of **EP02** that show p···p interactions, connecting the hydrogen bond chains represented in different colors (Symmetry code: (iii) −x + 1, −y + 1, −z + 1) with interactions shown as black dashed lines; Figure S15: View of the asymmetric unit of **EP03** with the numbering scheme of molecular structure, where atomic displacement ellipsoids are drawn at the 50% probability level; Figure S16: Perspective view of **EP03** showing the weak C(sp3)–H···N chain [symmetry code (iv) x + 1, y − 1, z] propagating along the [1−10] direction, and the secondary p···p stacking interactions between benzodioxole rings [symmetry code (v) −x + 2, −y + 1, −z + 1] that connect these chains into layers, with relevant contacts shown as dashed lines; Figure S17: View of the asymmetric unit of **EP04** with the numbering scheme of molecular structure, where atomic displacement ellipsoids are drawn at the 50% probability level; Figure S18: Perspective view of **EP04** showing the two independent p···p stacking contacts between the pendant 4–chlorophenyl ring and both rings of a symmetry–related quinoline system [symmetry code (vi) −x + 1, −y + 1, −z + 1], generating discrete centrosymmetric dimers, with relevant contacts shown as dashed lines; Table S1: Spin densities (ρspin) of the radical anionic (RA) and radical cationic (RC) species, and condensed Fukui functions *f*^+^ and *f*^−^ for the numbered atoms of the compound **EP01**, calculated at the M06–2X/def2–TZVPP level using the SMD implicit solvation model in methanol (A.N.: atom number); Table S2: Spin densities (ρspin) of the radical anionic (RA) and radical cationic (RC) species, and condensed Fukui functions *f*^+^ and *f*^−^ for the numbered atoms of the compound **EP02**, calculated at the M06–2X/def2–TZVPP level using the SMD implicit solvation model in methanol (A.N.: atom number); Table S3: Spin densities (ρspin) of the radical anionic (RA) and radical cationic (RC) species, and condensed Fukui functions *f*^+^ and *f*^−^ for the numbered atoms of the compound **EP03**, calculated at the M06–2X/def2–TZVPP level using the SMD implicit solvation model in methanol (A.N.: atom number); Table S4**:** Spin densities (ρspin) of the radical anionic (RA) and radical cationic (RC) species, and condensed Fukui functions *f*^+^ and *f*^−^ for the numbered atoms of the compound **EP04**, calculated at the M06–2X/def2–TZVPP level using the SMD implicit solvation model in methanol (A.N.: atom number); Table S5: Cartesian coordinates (.xyz) of the optimized structure of compound **EP01** calculated at the M06–2X/def2–TZVPP level using the SMD implicit solvation model in methanol (Number of atoms: 42); Table S6: Cartesian coordinates (.xyz) of the optimized structure of compound **EP02** calculated at the M06–2X/def2–TZVPP level using the SMD implicit solvation model in methanol (Number of atoms: 40); Table S7: Cartesian coordinates (.xyz) of the optimized structure of compound **EP03** calculated at the M06–2X/def2–TZVPP level using the SMD implicit solvation model in methanol (Number of atoms: 34); Table S8: Cartesian coordinates (.xyz) of the optimized structure of compound **EP04** calculated at the M06–2X/def2–TZVPP level using the SMD implicit solvation model in methanol (Number of atoms: 30); Table S9: Crystal data, data collection, and structure refinement for **EP01, EP02, EP03** and **EP04** compounds; Table S10: Geometric parameters of the relevant intermolecular interactions in **EP01**–**EP04**. Refs. [16,41,42] are cited in the Supplementary Materials.
