# Supplementary material for: Insights into Structure–Property Relationships in Quinoline–Based D–π–A Systems: A Synthetic and Computational Approach
Source: Molecules. 2026 Oct 19;31(20):3753. doi: 10.3390/molecules31203753 (PMC13708009; doi:10.3390/molecules31203753)
Supplement: Supplementary file 1 [file molecules-31-03753-s001.zip › molecules-4525187-supplementary.pdf]

Supporting_Information

Article

Insights into Structure–Property Relationships in Quinoline–Based D–π–A Systems: A Synthetic and Computational Approach

Cristian Rojas-Peña [1], Roger Espinosa-Saez [2,*], Camilo Guzmán-Terán [2], Claudio A. Jiménez [3], Angie Ortega Villadiego [4], Jairo Enrique Salazar [4], Dayanna Orosco [3], Yeray A. Rodríguez-Núñez [5], Jhon J. López [6], Carlos Mario Meléndez [4,*] and Efraín Polo-Cuadrado [3,*]

[1] Laboratorio de Radicales Libres y Antioxidantes, Facultad de Ciencias Químicas y Farmacéuticas,
Universidad de Chile, Av. Libertador Bernardo O'Higgins 1058, Santiago 8330111, Chile; cristian.rojas.p@ug.uchile.cl

[2] Grupo de Investigación IDEFARMA, Departamento de Regencia y Farmacia, Facultad Ciencias de la Salud, Universidad de Córdoba, Monteria 230002, Colombia; cantonioguzman@correo.unicordoba.edu.co

[3] Departamento de Química Orgánica, Facultad de Ciencias Químicas, Universidad de Concepción,
Concepcion 4130000, Chile; cjimenez@udec.cl (C.A.J.); dorosco2026@udec.cl (D.O.)

[4] Faculty of Basic Science, Molecular Biology Research Laboratory, Organic and Biomedical Chemistry
Research Group, Universidad Del Atlantico, Barranquilla 081007, Colombia; angiecortega@mail.uniatlantico.edu.co (A.O.V.); jairosalazar@mail.uniatlantico.edu.co (J.E.S.)

[5] Laboratorio de Síntesis y Reactividad de Compuestos Orgánicos, Facultad de Ciencias Exactas,
Departamento de Ciencias Químicas, Universidad Andrés Bello, Santiago 8370146, Chile; yeray.rodriguez@unab.cl

[6] Departamento de Química Orgánica, Facultad de Química y de Farmacia, Escuela de Química, Pontificia Universidad Católica de Chile, Santiago 7820436, Chile; jjlopez@uc.cl

* Correspondence: rdespinosasaez@correo.unicordoba.edu.co (R.E.-S.); carlosmelendez@mail.uniatlantico.edu.co (C.M.M.); epolo@udec.cl (E.P.-C.)

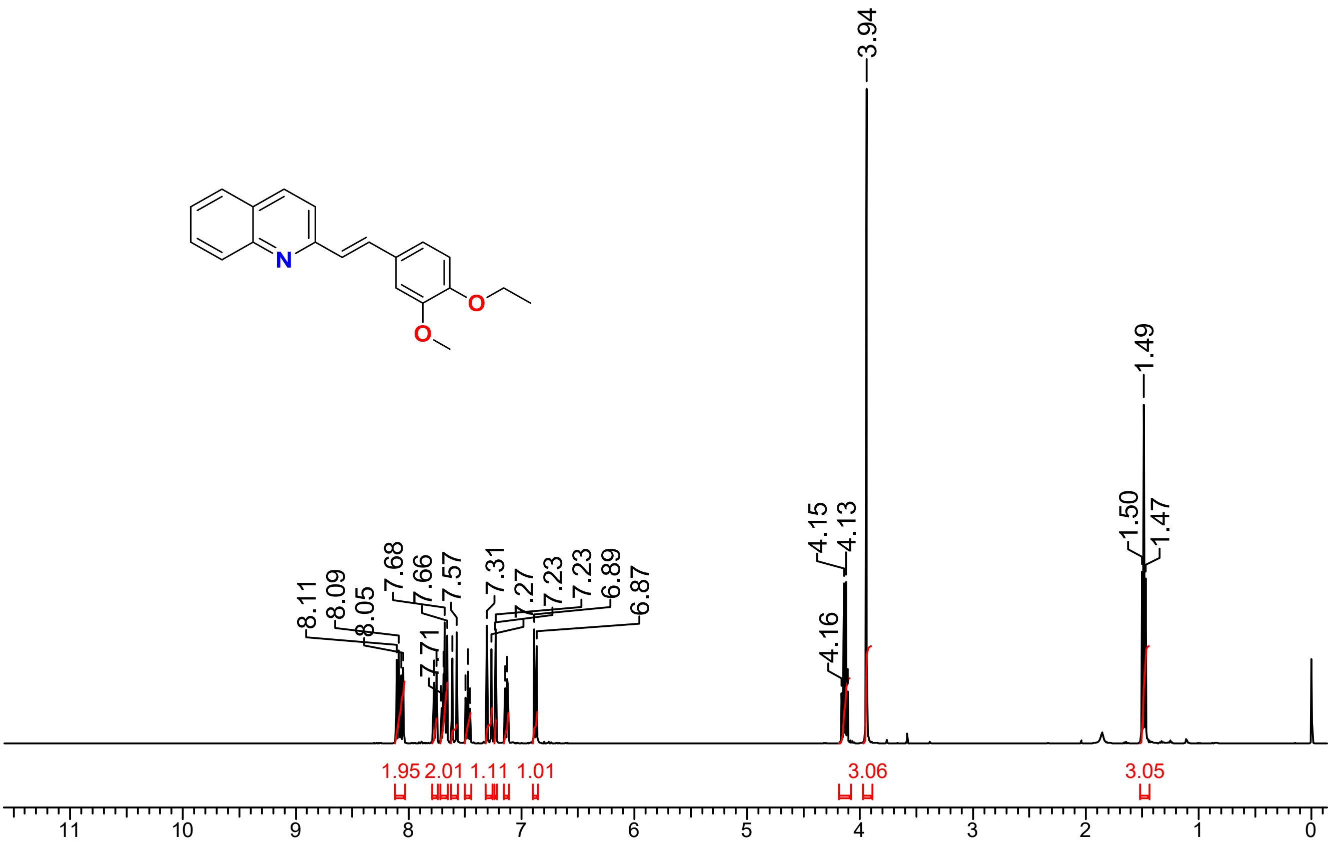

Figure S1. ^{1}H-NMR compound **EP01**.

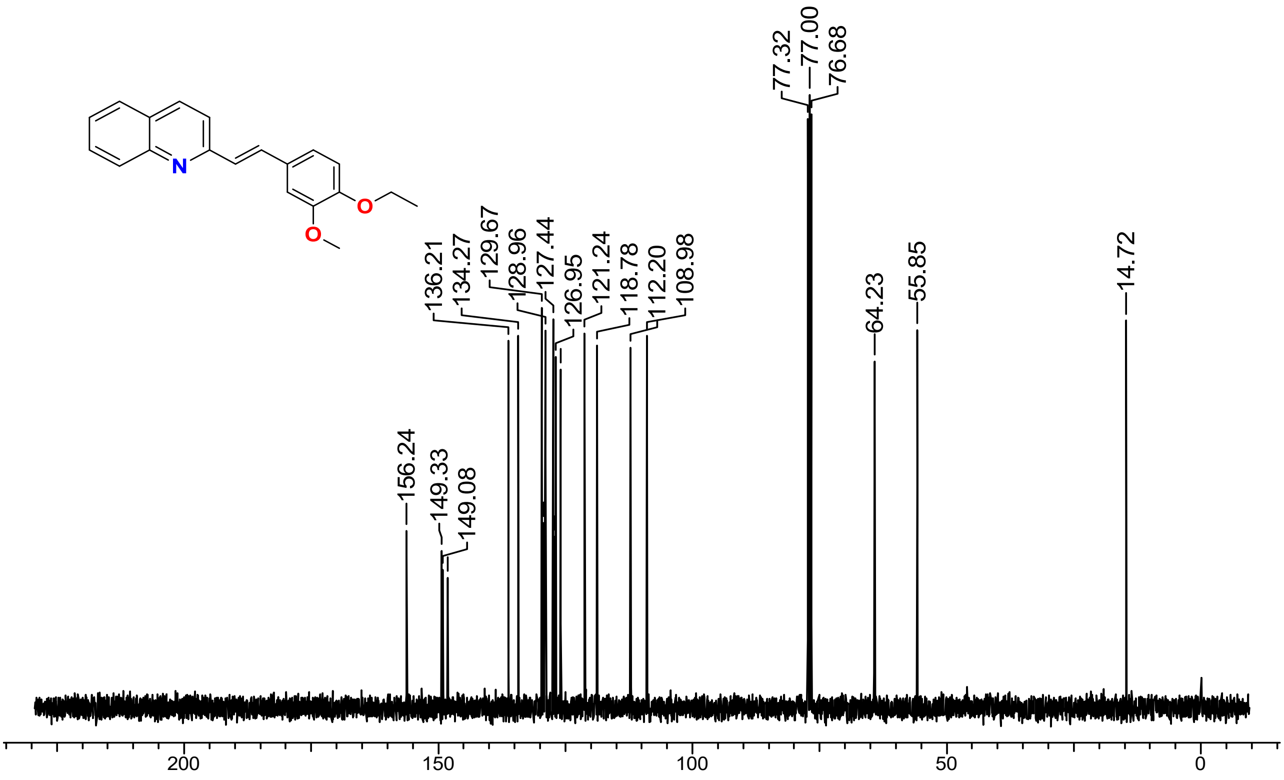

Figure S2. ^{13}C-NMR compound **EP01**.

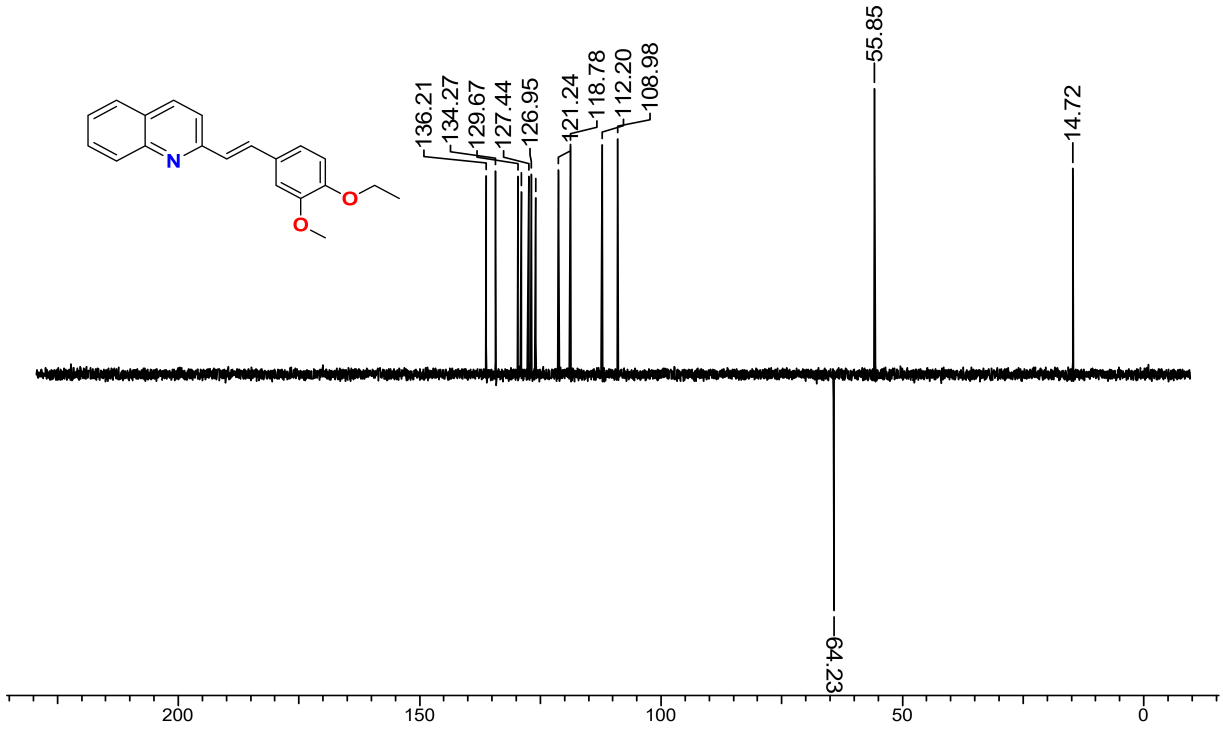

Figure S3. *Dept-135 compound **EP01**.*

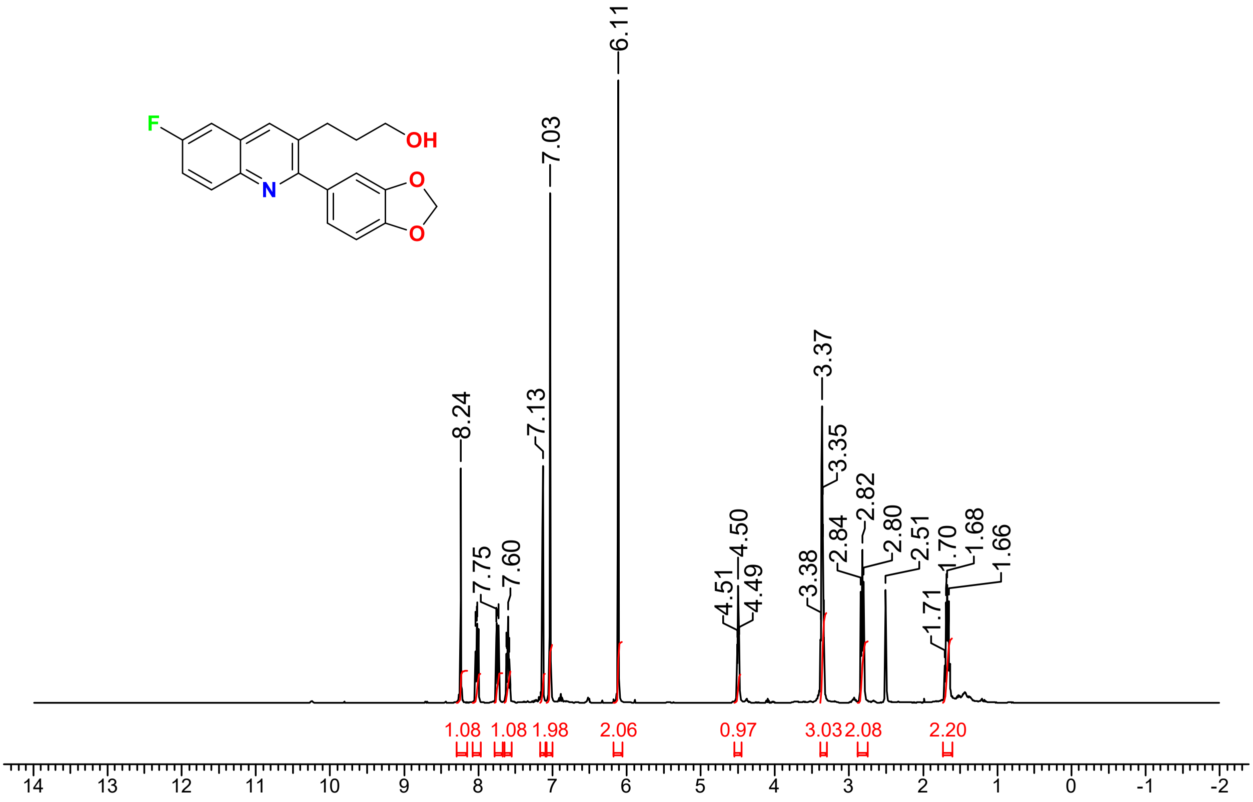

Figure S4. *^{1}H-NMR compound **EP02.***

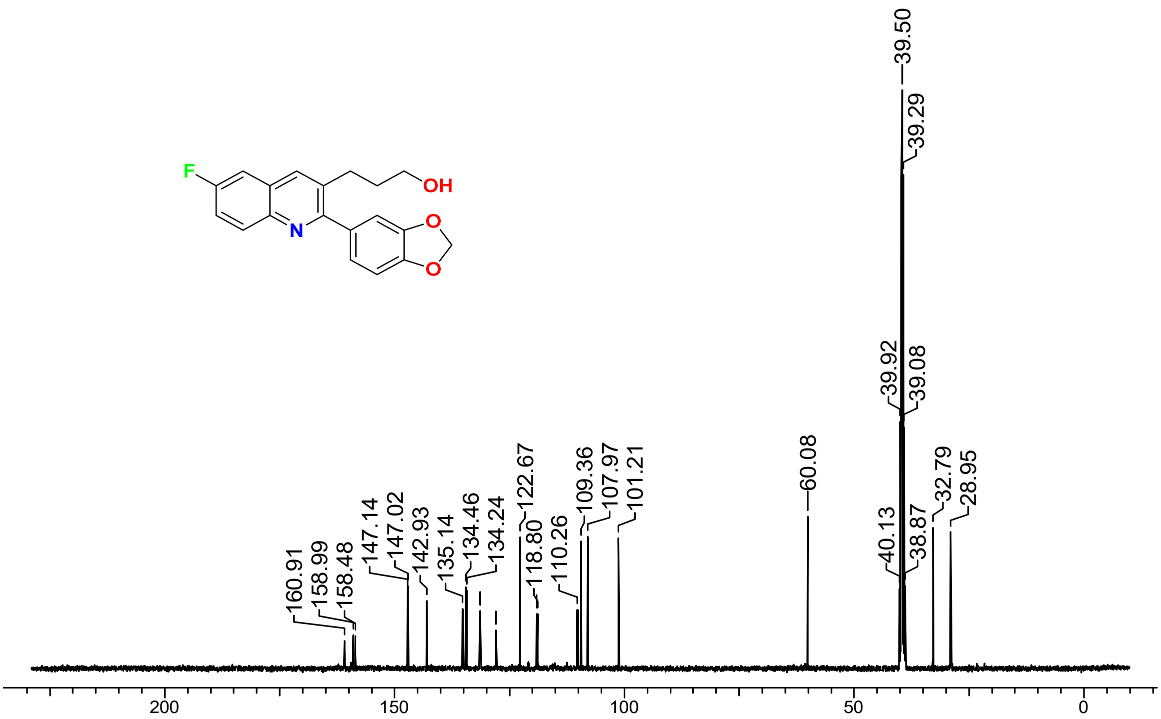

Figure S5. ^{13}C-NMR compound ***EP02.***

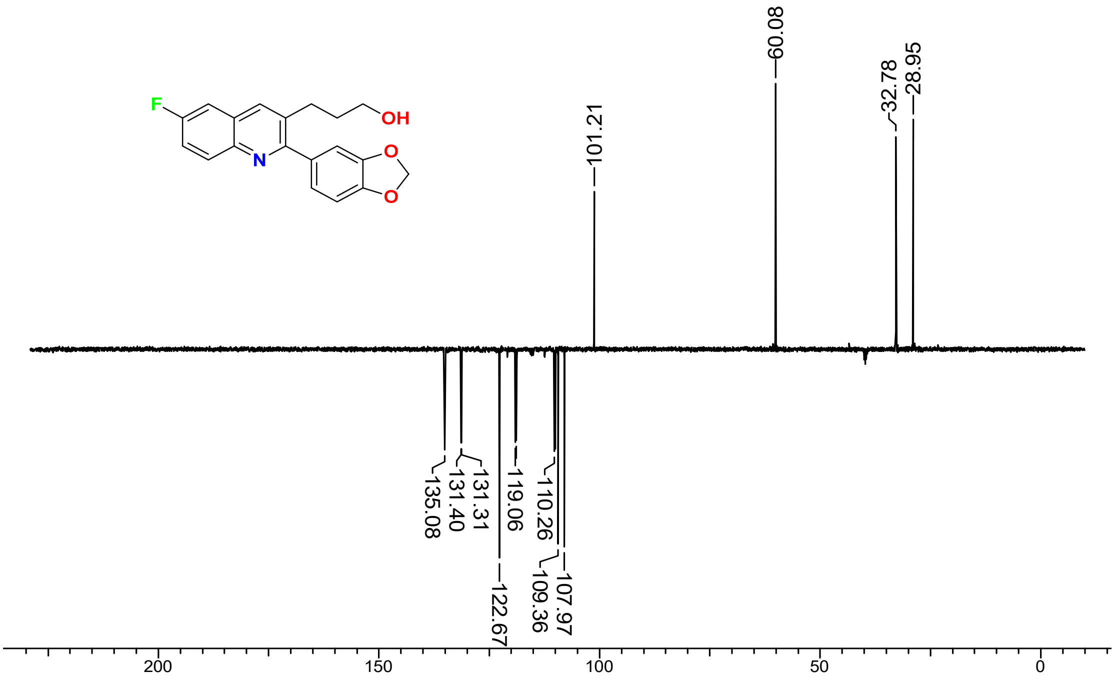

Figure S6. *Dept 135 compound* ***EP02***.

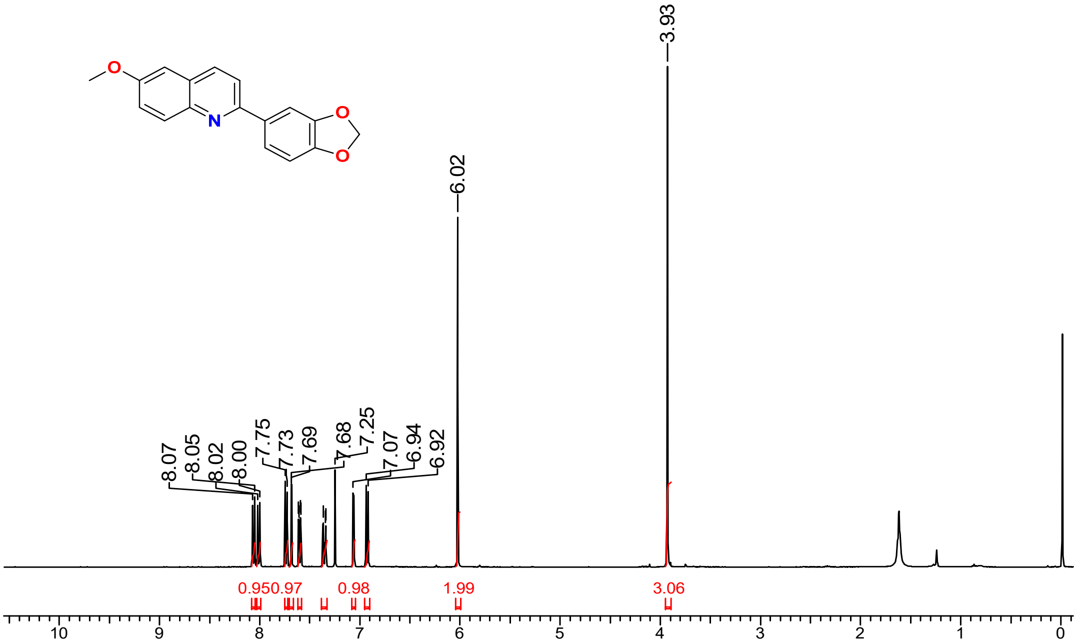

Figure S7. *¹H-NMR compound* ***EP03***

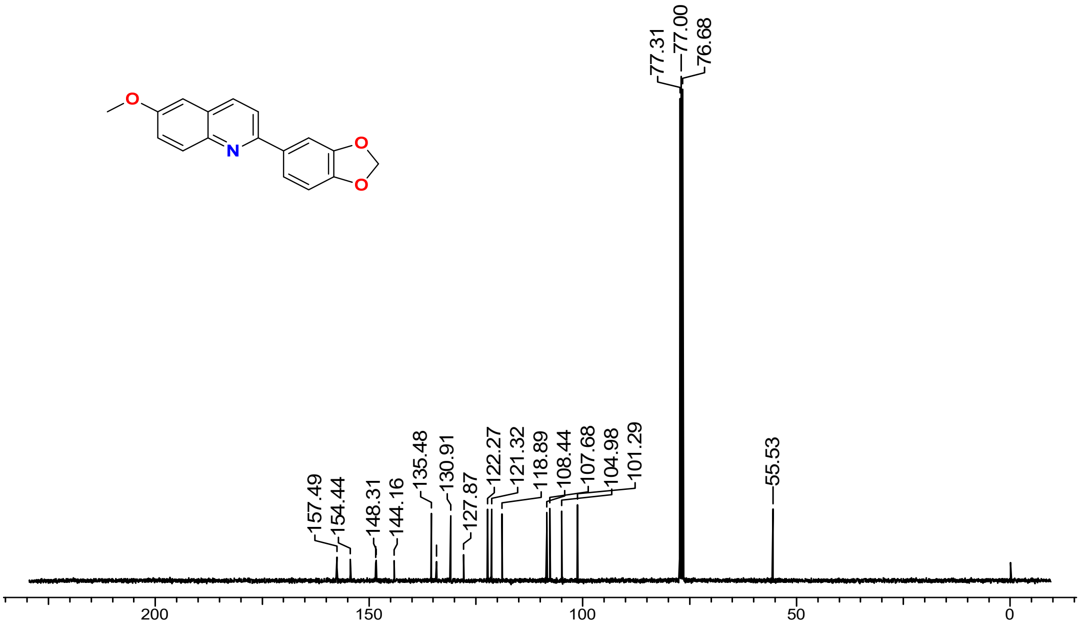

Figure S8. *¹³C-NMR compound* ***EP03***.

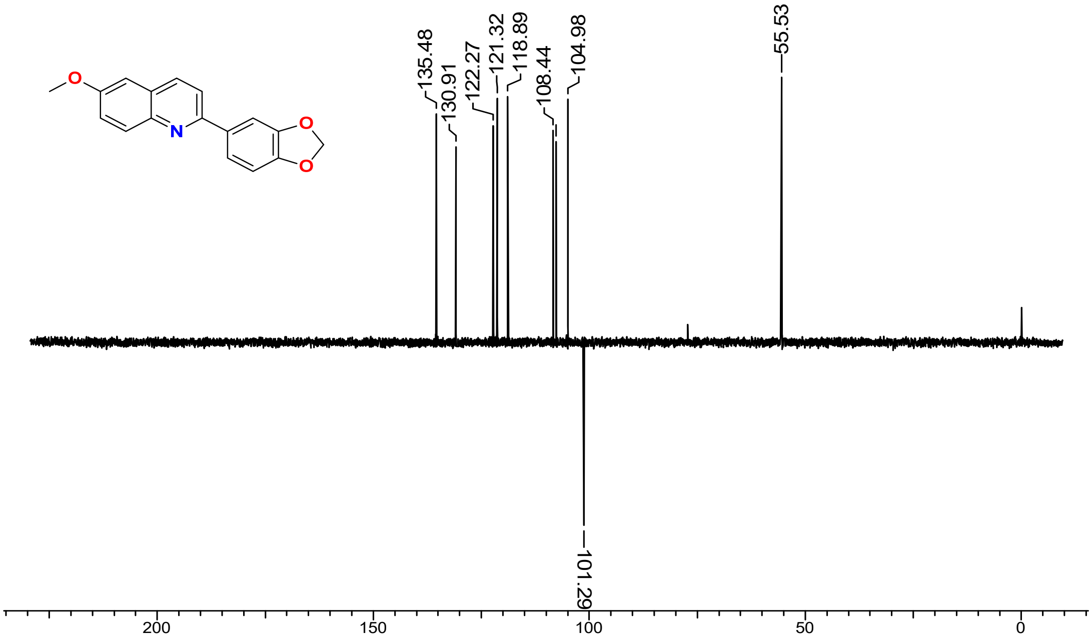

Figure S9. *Dept 135 compound* ***EP03***.

***Table S1.** Spin densities (ρ_{spin}) of the radical anionic (RA) and radical cationic (RC) species, and condensed Fukui functions f^{+} and f^{-} for the numbered atoms of the compound **EP01**. Calculated at the M06-2X/def2-TZVPP level using the SMD implicit solvation model in methanol. A.N.: atom number.*

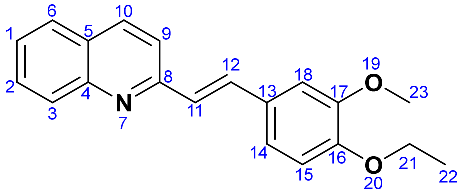

A.N.	ρ_{spin}(RA)	ρ_{spin}(RC)	f^{+}	f^{-}
1	0.07	0.02	0.05	0.02
2	0.01	0.00	0.03	0.01
3	0.06	0.01	0.04	0.01
4	0.00	0.00	0.02	0.01
5	0.03	0.02	0.03	0.01
6	0.02	-0.01	0.03	0.01
7	0.16	0.05	0.09	0.03
8	0.13	-0.01	0.08	0.01
9	0.00	0.02	0.03	0.01
10	0.07	-0.01	0.06	0.01
11	0.05	0.15	0.06	0.08
12	0.18	0.01	0.10	0.03
13	0.01	0.14	0.02	0.08
14	0.06	0.12	0.04	0.08
15	-0.01	0.01	0.02	0.05
16	0.06	0.18	0.03	0.09
17	-0.01	0.09	0.01	0.06
18	0.04	-0.03	0.03	0.03
19	0.00	0.06	0.01	0.05
20	0.02	0.13	0.01	0.08
21	0.00	0.00	0.00	0.01
22	0.00	0.00	0.00	0.01
23	0.00	0.00	0.00	0.01

***Table S2.** Spin densities (ρ_{spin}) of the radical anionic (RA) and radical cationic (RC) species, and condensed Fukui functions f^{+} and f^{-} for the numbered atoms of the compound **EP02**. Calculated at the M06-2X/def2-TZVPP level using the SMD implicit solvation model in methanol. A.N.: atom number.*

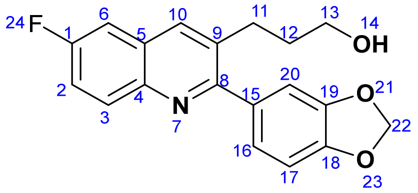

A.N.	ρ_{spin}(RA)	ρ_{spin}(RC)	f^{+}	f^{-}
1	0.05	0.04	0.04	0.04
2	0.08	0.00	0.07	0.03
3	0.12	0.02	0.06	0.03
4	0.01	0.02	0.01	0.03

5	0.00	0.02	0.01	0.02
6	0.08	0.00	0.06	0.02
7	0.19	0.03	0.05	0.05
8	0.10	0.02	0.06	0.00
9	0.06	0.02	0.05	0.00
10	0.17	-0.01	0.09	0.01
11	0.01	0.01	0.01	0.00
12	0.01	0.00	0.01	0.00
13	0.00	0.00	0.01	0.00
14	0.00	0.00	-0.03	0.05
15	0.01	0.13	-0.02	0.08
16	0.02	0.14	0.01	0.08
17	0.00	0.00	0.02	0.04
18	0.01	0.17	0.02	0.08
19	0.00	0.11	0.01	0.07
20	0.01	-0.02	-0.01	0.04
21	0.00	0.10	-0.01	0.09
22	0.00	0.01	0.02	0.03
23	0.00	0.14	0.00	0.10
24	0.01	0.01	0.03	0.04

Table S3. *Spin densities (ρ_{spin}) of the radical anionic (RA) and radical cationic (RC) species, and condensed Fukui functions f^+ and f^- for the numbered atoms of the compound* ***EP03****. Calculated at the M06-2X/def2-TZVPP level using the SMD implicit solvation model in methanol. A.N.: atom number.*

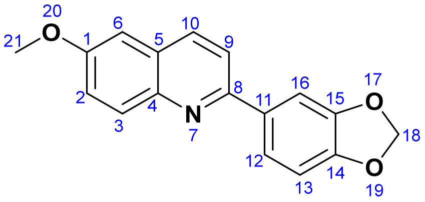

A.N.	$\boldsymbol{\rho_{spin}}$(RA)	$\boldsymbol{\rho_{spin}}$(RC)	f^+	f^-
1	0.04	0.03	0.04	0.02
2	0.08	0.00	0.06	0.01
3	0.11	0.02	0.07	0.02
4	0.01	0.01	0.03	0.02
5	0.00	0.02	0.03	0.02
6	0.07	-0.01	0.06	0.01
7	0.19	0.04	0.10	0.03
8	0.11	0.00	0.08	0.01
9	0.05	0.02	0.06	0.01
10	0.18	-0.01	0.10	0.01
11	0.01	0.13	0.01	0.08
12	0.03	0.13	0.02	0.09
13	0.00	0.01	0.01	0.05
14	0.03	0.17	0.02	0.09
15	0.00	0.12	0.01	0.07
16	0.03	-0.03	0.02	0.04

17	0.00	0.11	0.01	0.08
18	0.00	0.01	0.00	0.03
19	0.01	0.16	0.01	0.10
20	0.01	0.02	0.02	0.01
21	0.00	0.00	0.01	0.00

Table S4. *Spin densities (ρ_{spin}) of the radical anionic (RA) and radical cationic (RC) species, and condensed Fukui functions f^+ and f^- for the numbered atoms of the compound* ***EP04****. Calculated at the M06-2X/def2-TZVPP level using the SMD implicit solvation model in methanol. A.N.: atom number.*

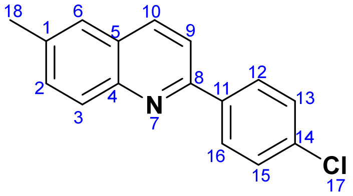

A.N.	ρ_{spin}(RA)	ρ_{spin}(RC)	f^+	f^-
1	0.05	0.22	0.04	0.11
2	0.05	-0.03	0.05	0.03
3	0.08	0.16	0.06	0.09
4	0.01	0.09	0.03	0.06
5	0.01	0.02	0.03	0.03
6	0.06	0.18	0.05	0.11
7	0.20	0.07	0.10	0.05
8	0.14	0.08	0.08	0.05
9	0.02	-0.02	0.05	0.03
10	0.17	0.06	0.10	0.05
11	0.03	0.01	0.02	0.01
12	0.04	0.03	0.03	0.02
13	0.00	0.00	0.02	0.01
14	0.05	0.03	0.03	0.02
15	0.00	0.00	0.02	0.01
16	0.03	0.02	0.03	0.02
17	0.01	0.01	0.03	0.02
18	0.01	0.02	0.01	0.03

Table S5. *Cartesian coordinates (.xyz) of the optimized structure of compound* ***EP01*** *Calculated at the M06-2X/def2-TZVPP level using the SMD implicit solvation model in methanol. Number of atoms: 42.*

No.	Atom	X (Å)	Y (Å)	Z (Å)
1	C	7.417863	2.130285	0.239123
2	C	7.338138	0.720699	0.241945
3	C	6.123424	0.090252	0.208978
4	C	4.9267	0.84427	0.171379
5	C	5.00986	2.256598	0.167033
6	C	6.275036	2.882942	0.201649
7	N	3.736031	0.185235	0.140583
8	C	2.616839	0.881839	0.102937

9	C	2.611762	2.30675	0.09526
10	C	3.792467	2.979066	0.127898
11	C	1.378941	0.098567	0.070118
12	C	0.139918	0.599429	0.009057
13	C	-1.103	-0.17538	-0.03068
14	C	-1.14264	-1.56516	-0.04027
15	C	-2.3561	-2.2421	-0.08111
16	C	-3.55282	-1.54144	-0.11361
17	C	-3.52777	-0.1334	-0.10361
18	C	-2.3166	0.530208	-0.06344
19	O	-4.74382	0.47584	-0.13744
20	O	-4.78349	-2.10975	-0.15797
21	C	-4.8564	-3.53707	-0.18333
22	C	-6.31314	-3.91687	-0.25044
23	C	-4.75929	1.8957	-0.13231
24	H	8.386343	2.611247	0.266797
25	H	8.248199	0.135963	0.269074
26	H	6.052299	-0.98954	0.210835
27	H	6.320736	3.965029	0.198461
28	H	1.677459	2.847857	0.066184
29	H	3.819094	4.061899	0.124368
30	H	1.535972	-0.97326	0.096531
31	H	-0.00069	1.67468	-0.01517
32	H	-0.22976	-2.14434	-0.02026
33	H	-2.35855	-3.3219	-0.08903
34	H	-2.28972	1.610874	-0.05701
35	H	-4.38529	-3.93599	0.717722
36	H	-4.31054	-3.90699	-1.05395
37	H	-6.85043	-3.53918	0.619077
38	H	-6.4053	-5.00223	-0.26867
39	H	-6.77494	-3.51606	-1.15237
40	H	-5.8052	2.185803	-0.1657
41	H	-4.24082	2.2932	-1.00604
42	H	-4.30077	2.286428	0.777406

Table S6. *Cartesian coordinates (.xyz) of the optimized structure of compound* ***EP02*** *Calculated at the M06-2X/def2-TZVPP level using the SMD implicit solvation model in methanol. Number of atoms: 40.*

No.	Atom	X (Å)	Y (Å)	Z (Å)
1	C	-2.16836	-0.7268	-0.12923
2	C	-2.24591	0.674527	-0.07735
3	C	-1.09104	1.394346	0.04888
4	C	0.15971	0.736628	0.122945
5	C	0.203429	-0.67387	0.064915
6	C	-0.99573	-1.41152	-0.06269
7	N	1.288825	1.483315	0.251189
8	C	2.456252	0.886964	0.317063

9	C	2.611984	-0.53587	0.234262
10	C	1.474857	-1.28542	0.115379
11	C	3.961879	-1.19928	0.163288
12	C	4.64697	-0.95389	-1.18246
13	C	6.023756	-1.58761	-1.22355
14	O	6.678242	-1.37205	-2.46972
15	C	3.634124	1.790049	0.458834
16	C	4.507138	1.666268	1.533105
17	C	5.596023	2.530236	1.699948
18	C	5.761341	3.5038	0.748898
19	C	4.895036	3.633325	-0.32602
20	C	3.821529	2.800175	-0.5007
21	O	5.331474	4.655717	-1.13544
22	C	6.296328	5.357959	-0.35167
23	O	6.756982	4.443722	0.644249
24	F	-3.32509	-1.40613	-0.25419
25	H	-3.21365	1.153165	-0.13954
26	H	-1.11371	2.474846	0.092695
27	H	-0.97932	-2.49224	-0.11041
28	H	1.543044	-2.36469	0.039492
29	H	3.824495	-2.27234	0.30366
30	H	4.61403	-0.85506	0.965371
31	H	4.742765	0.118143	-1.37001
32	H	4.033132	-1.37368	-1.98437
33	H	5.951999	-2.65936	-1.01564
34	H	6.665666	-1.14254	-0.46228
35	H	6.155169	-1.79749	-3.15706
36	H	4.332698	0.899653	2.276122
37	H	6.270383	2.436173	2.538963
38	H	3.154056	2.904421	-1.34461
39	H	5.813731	6.206942	0.136696
40	H	7.128176	5.659082	-0.97953

***Table* S7.** Cartesian coordinates (.xyz) of the optimized structure of compound **EP03**. Calculated at the M06-2X/def2-TZVPP level using the SMD implicit solvation model in methanol. Number of atoms: 34.

No.	Atom	X (Å)	Y (Å)	Z (Å)
1	C	-5.222336	-1.913196	0.053255
2	C	-5.347258	-0.505757	-0.022496
3	C	-4.235753	0.287819	0.080916
4	C	-2.953221	-0.273154	0.263911
5	C	-2.840211	-1.681952	0.345140
6	C	-3.987206	-2.487662	0.236077
7	N	-1.877075	0.554298	0.356177
8	C	-0.683311	0.037833	0.527943
9	C	-0.474866	-1.369614	0.615211
10	C	-1.538413	-2.212485	0.525748
11	C	0.466041	0.974476	0.607881

12	C	1.627779	0.626801	1.290745
13	C	2.721430	1.495478	1.382225
14	C	2.599960	2.710544	0.758465
15	C	1.444420	3.068432	0.079356
16	C	0.364368	2.234173	-0.015016
17	O	1.594094	4.320920	-0.445427
18	C	2.903246	4.766551	-0.085454
19	O	3.509292	3.722658	0.682388
20	O	-6.281575	-2.757236	-0.047010
21	H	-6.315578	-0.047825	-0.162434
22	H	-4.324731	1.365023	0.021565
23	H	-3.900162	-3.565701	0.294668
24	H	-1.403789	-3.286067	0.579216
25	H	1.694028	-0.330682	1.788367
26	H	3.617506	1.222681	1.921721
27	H	-0.526373	2.521086	-0.554967
28	H	2.828704	5.664931	0.525229
29	H	3.488736	4.938189	-0.987387
30	H	0.524869	-1.763933	0.726249
31	C	-7.573831	-2.199987	-0.242079
32	H	-7.618306	-1.628027	-1.170227
33	H	-8.255940	-3.043433	-0.303245
34	H	-7.857106	-1.564613	0.598759

***Table* S8.** Cartesian coordinates (.xyz) of the optimized structure of compound **EP04**. Calculated at the M06-2X/def2-TZVPP level using the SMD implicit solvation model in methanol. Number of atoms: 30.

No.	Atom	X (Å)	Y (Å)	Z (Å)
1	C	3.7886523333	0.9633926333	0.2012467000
2	C	3.5870063333	-0.4210623667	0.4492327000
3	C	2.3411333333	-0.9794793667	0.4261037000
4	C	1.2043163333	-0.1794333667	0.1509517000
5	C	1.3927993333	1.2001616333	-0.0971653000
6	C	2.6975793333	1.7469546333	-0.0667763000
7	N	-0.0237276667	-0.7600023667	0.1397957000
8	C	-1.0788836667	-0.0173113667	-0.1092133000
9	C	-0.9832856667	1.3732346333	-0.3814683000
10	C	0.2418263333	1.9709036333	-0.3738613000
11	C	-2.4034106667	-0.6897793667	-0.1095233000
12	C	-2.5070996667	-2.0382403667	-0.4519253000
13	C	-3.7347516667	-2.6797893667	-0.4585163000
14	C	-4.8704356667	-1.9646443667	-0.1090173000
15	C	-4.7972586667	-0.6268303667	0.2428737000
16	C	-3.5624836667	0.0042186333	0.2364177000
17	H	4.4497703333	-1.0413023667	0.6633547000
18	H	2.1942723333	-2.0352043667	0.6160987000
19	H	2.8196913333	2.8070296333	-0.2593103000

20	H	-1.8712946667	1.9428656333	-0.6152943000
21	H	0.3465663333	3.0285116333	-0.5845253000
22	H	-1.6179466667	-2.5889613667	-0.7269863000
23	H	-3.8093626667	-3.7223823667	-0.7357803000
24	H	-3.5116196667	1.0448816333	0.5280187000
25	H	-5.6896036667	-0.0841483667	0.5229287000
26	Cl	-6.4144526667	-2.7628393667	-0.1120013000
27	C	5.1797463333	1.5242376333	0.2434557000
28	H	5.8245503333	1.0134806333	-0.4742623000
29	H	5.1838193333	2.5887166333	0.0143147000
30	H	5.6238873333	1.3828216333	1.2308337000

Crystal Structure Description

Appropriate single crystals of **EP01** were subjected to X-ray analysis at room temperature; the resulting molecular structure is illustrated in Figure 10S. **EP01** crystallizes in the non-centrosymmetric orthorhombic space group $P2_12_12_1$, with one independent molecule constituting the asymmetric unit. Inspection of the crystal packing reveals that the dominant intermolecular interaction consists of p···p stacking contacts between quinoline ring systems related by translational symmetry [symmetry code: (i) *x*, *y*–1, *z*], propagating along the crystallographic *b*-axis, which assemble into one-dimensional supramolecular chains (Figure 11S).

EP02 crystallizes in the centrosymmetric monoclinic space group $P2_1/n$, with one independent molecule constituting the asymmetric unit (Figure 12S). The crystal packing is dominated by an O–H···N hydrogen bond established between the hydroxyl group of the propanolic side chain and the N1 atom of a symmetry-related molecule [symmetry code: (ii) *x*+1/2, −*y*+3/2, *z*−1/2], propagating along the [101] direction and generating one-dimensional supramolecular chains with an ABAB pattern among each molecule (Figure 13S). These hydrogen-bonded chains are, in turn, connected to one another through p···p stacking interactions between quinoline rings related by a crystallographic inversion centre located at the midpoint of the unit cell [symmetry code: (iii) −*x*+1, −*y*+1, −*z*+1], extending the hydrogen-bonded chains into two-dimensional supramolecular layers (Figure 14S).

Compound **EP03** crystallizes in the centrosymmetric triclinic space group *P*-1, with one independent molecule constituting the asymmetric unit (Figure 15S). As in **EP02**, the quinoline ring is directly bonded to the pendant aryl group with a benzo[d][1,3]dioxol-5-yl substituent, through a single C–C bond. Unlike **EP02**, however, **EP03** possesses no classical hydrogen-bond donor group; its supramolecular packing is instead governed by a weak, non-directional C(sp^3)–H···N interaction, of the type statistically validated for methyl donors by Taylor and Kennard [3], established between a hydrogen atom of the methoxy methyl group and the N1 atom of a symmetry-related molecule [symmetry code: (iv) *x*+1, *y*–1, *z*]. This weak contact propagates via pure translational symmetry along the [1−10] direction, generating one-dimensional supramolecular chains. These chains are, in turn, connected to one another through p···p stacking interactions between benzodioxole rings related by a crystallographic inversion centre [symmetry code: (v) -x+2, -y+1, -z+1], propagating predominantly along the crystallographic *c*-axis, a direction essentially

orthogonal to the C–H···N chains, and thereby extending the hydrogen-bonded chains into two-dimensional supramolecular layers (Figure 16S).

Finally, compound **EP04** crystallizes in the centrosymmetric monoclinic space group $P2_1/c$, with one independent molecule constituting the asymmetric unit (Figure 17S). In **EP04**, the quinoline ring is directly bonded to a 4-chlorophenyl substituent. Since **EP04** possesses no hydrogen-bond donor group, bearing only a chloro and a methyl substituent, the crystal packing is governed exclusively by p···p stacking interactions. The pendant 4-chlorophenyl ring engages in two independent offset p···p contacts with both rings of a symmetry-related quinoline system across a crystallographic inversion centre [symmetry code: (vi) −*x*+1, −*y*+1, −*z*+1]. These two contacts generate discrete, centrosymmetric p-stacked dimers rather than an extended one-dimensional stacking motif, in contrast to the propagating chains observed in **EP01**. Beyond these discrete dimers, the molecules of **EP04** pack in a classic herringbone arrangement, characteristic of aromatic systems (Figure 18S).

A general descripción for four compound show a quinoline core essentially invariant across the series, as confirmed for **EP01** and **EP02** by a least-squares overlay of the bicyclic quinoline core (atoms N1, C1–C9; RMSD = 0.045 Å direct, 0.023 Å with inversion), the four compounds diverge markedly in the supramolecular synthon governing their crystal packing. The same overlay extended pairwise to **EP03** and **EP04** confirms this rigidity across the full series (direct/inversion RMSD ranges of 0.012–0.040 Å and 0.020–0.041 Å, respectively, for all six pairwise combinations), indicating that the bicyclic quinoline scaffold is essentially conformationally transferable irrespective of the substitution pattern at the 2- and 3-positions. Notably, the offset angle observed for the primary quinoline···quinoline stacking in **EP01** (28.03°) lies somewhat above the range reported for related quinoline- and isoquinoline-carboxamide systems (17.4–23.6°, corresponding to centroid-to-centroid separations of 3.657–3.851 Å), **[1,2]** including the closely analogous secondary p-stacking mode of the isoquinolinecarboxamide derivative, where (4,4)-net layers are linked through quinoline···quinoline contacts related by a crystallographic inversion centre,**[1]** a supramolecular hierarchy directly comparable to that observed here for **EP02**, suggesting an overall more slipped p-stacking arrangement in the present, amide-free quinoline systems than in those previously studied amide-linked homologues. **EP03** and **EP04** extend this structural comparison further: both share with **EP02** a direct biaryl linkage to the quinoline ring, in contrast to the vinylene bridge of **EP01**; **EP03**, however, packs via a weak C(sp^3)–H···N chain reinforced by p-stacking into layers, whereas **EP04**, lacking any hydrogen-bond donor, packs exclusively through p-stacked dimers in a herringbone arrangement,

illustrating how the nature and position of the peripheral substituents redirect the supramolecular hierarchy even when the biaryl connectivity to the quinoline core is conserved across **EP02**, **EP03** and **EP04**.

[1] Parra, N.; Guarda, L.; Belmar, J. B.; Hidalgo, P. I.; Jiménez, C. A.; Pasán, J.; Ruiz-Pérez, C. *CrystEngComm* **2013**, *15*, 7212–7221.

[2] Parra, N.; Belmar, J. B.; Jiménez, C. A.; Pasán, J.; Ruiz-Pérez, C. *CrystEngComm* **2017**, *19*, 1076–1088.

[3] Taylor, R.; Kennard, O. Crystallographic Evidence for the Existence of C–H···O, C–H···N, and C–H···Cl Hydrogen Bonds. *J. Am. Chem. Soc.* **1982**, 104, 5063–5070.

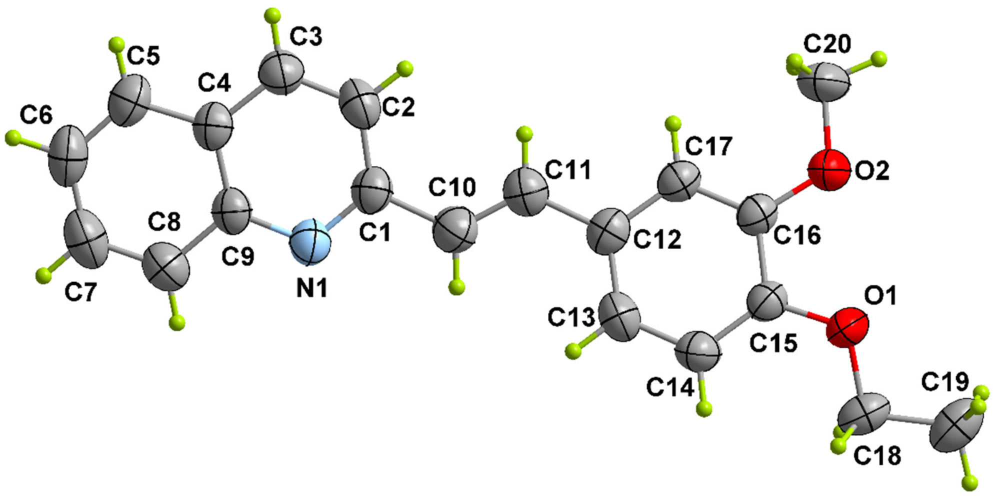

Figure S10. *View of the asymmetric unit of **EP01** with the numbering scheme of molecular structure. Atomic displacement ellipsoids are drawn at the 50% probability level.*

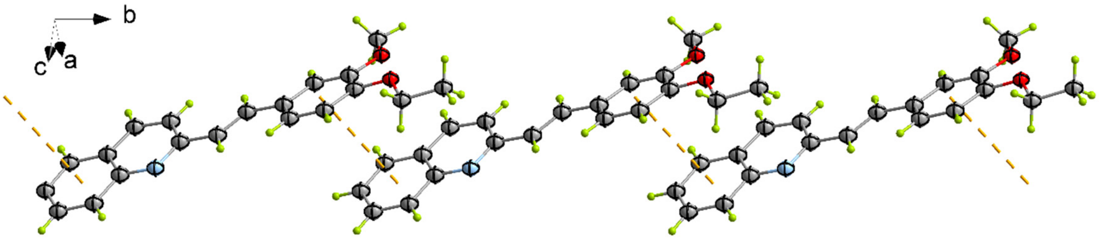

Figure S11. *Perspective view of* ***EP01*** *show the p ··· p interaction growing along the b-axis. The p···p interactions are shown as yellow dashed lines. (Symmetry code: (i) x, y–1, z).*

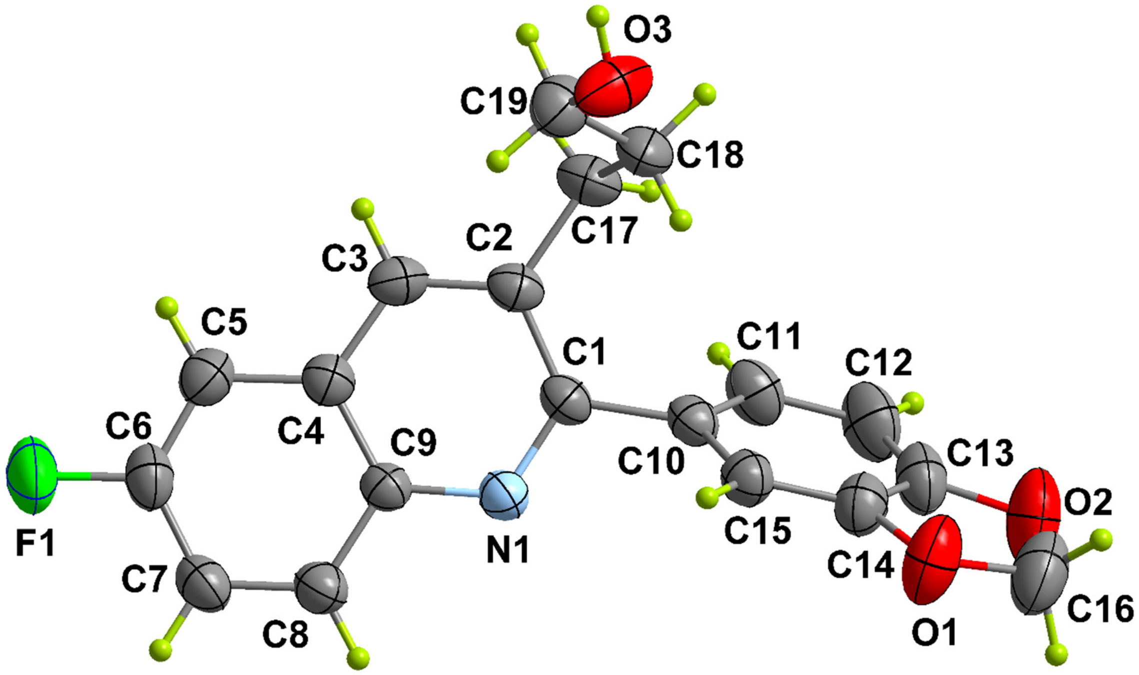

Figure S12. *View of the asymmetric unit of* ***EP02*** *with the numbering scheme of molecular structure. Atomic displacement ellipsoids are drawn at the 50% probability level.*

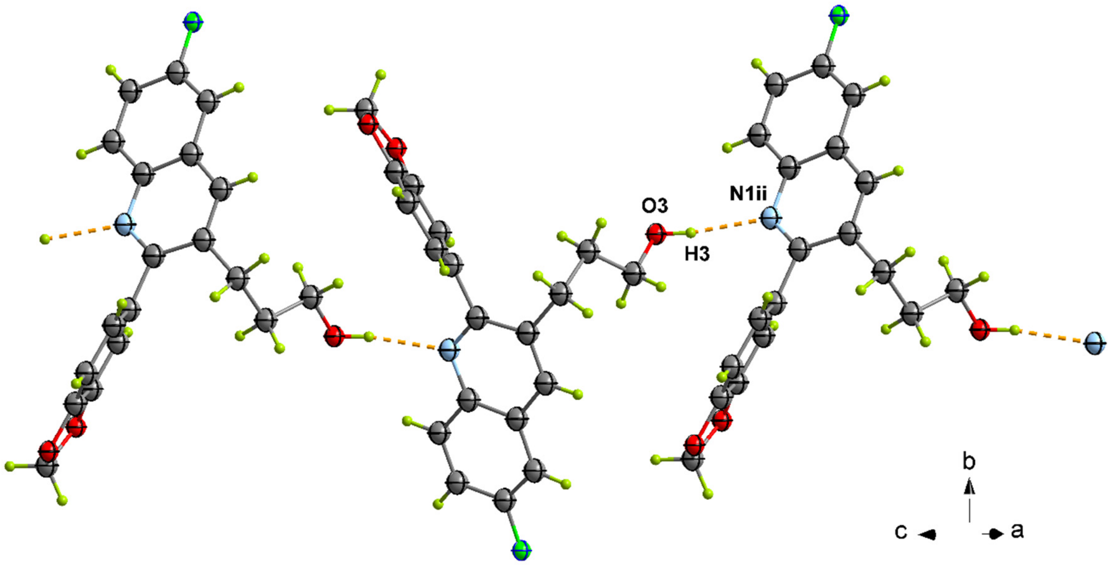

Figure S13. *Perspective view of* ***EP02*** *show the hydrogen bonding interaction growing along the [1,0,1] direction. The HBs interactions are shown as dashed lines. (Symmetry operation: (ii) x+1/2, −y+3/2, z−1/2).*

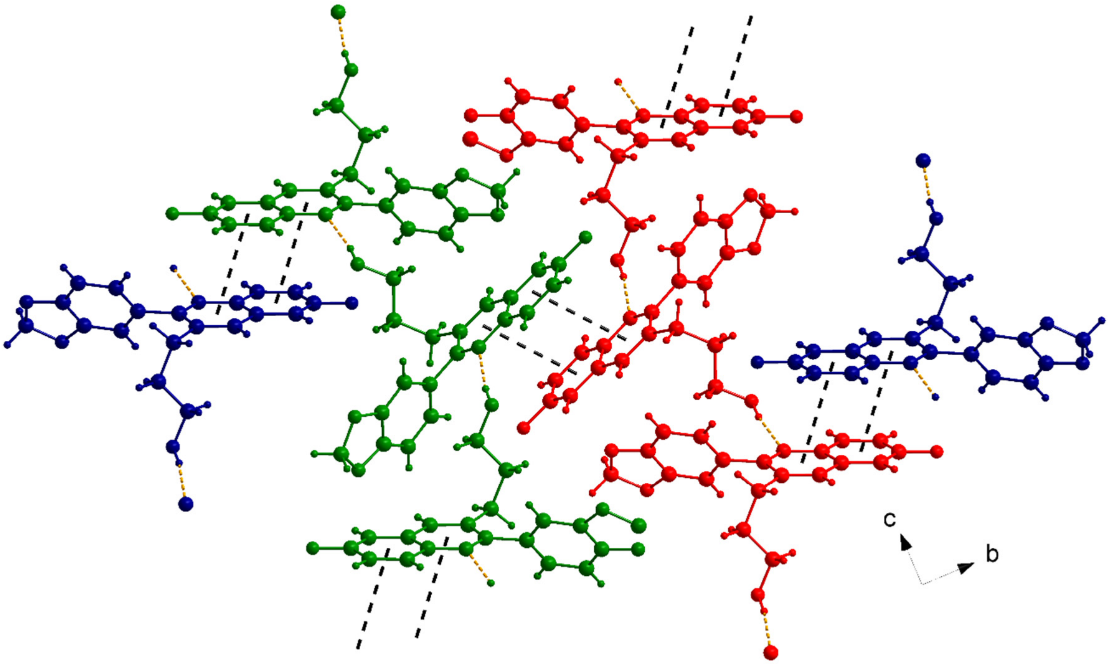

Figure S14. *View of ac-plane of* ***EP02*** *that show p···p interactions, connecting the hydrogen bond chains (represented in different colors). The p···p interactions are shown as black dashed lines. (Symmetry code: (iii) −x+1, −y+1, −z+1).*

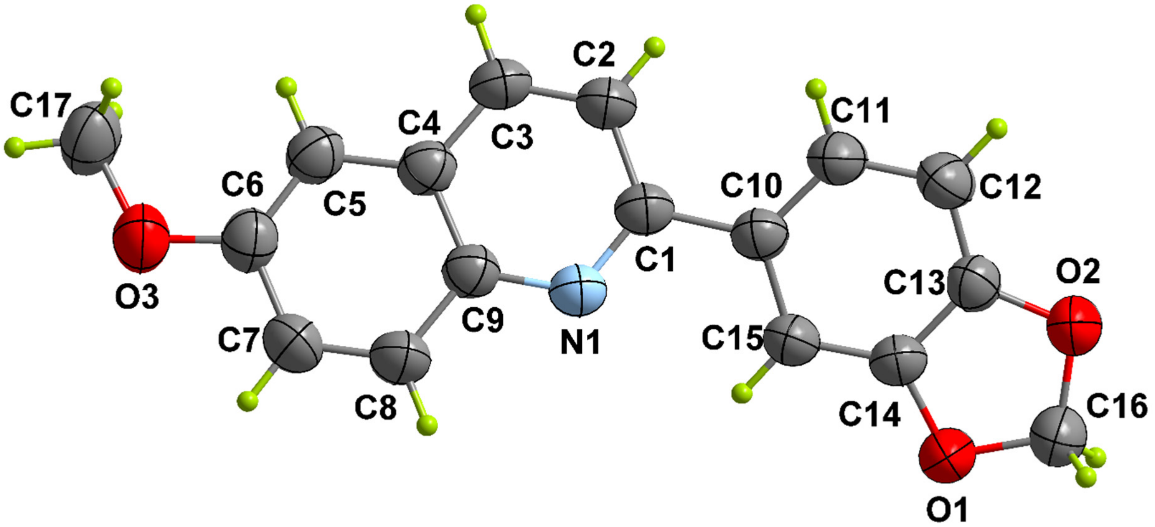

Figure S15. *View of the asymmetric unit of* ***EP03*** *with the numbering scheme of molecular structure. Atomic displacement ellipsoids are drawn at the 50% probability level.*

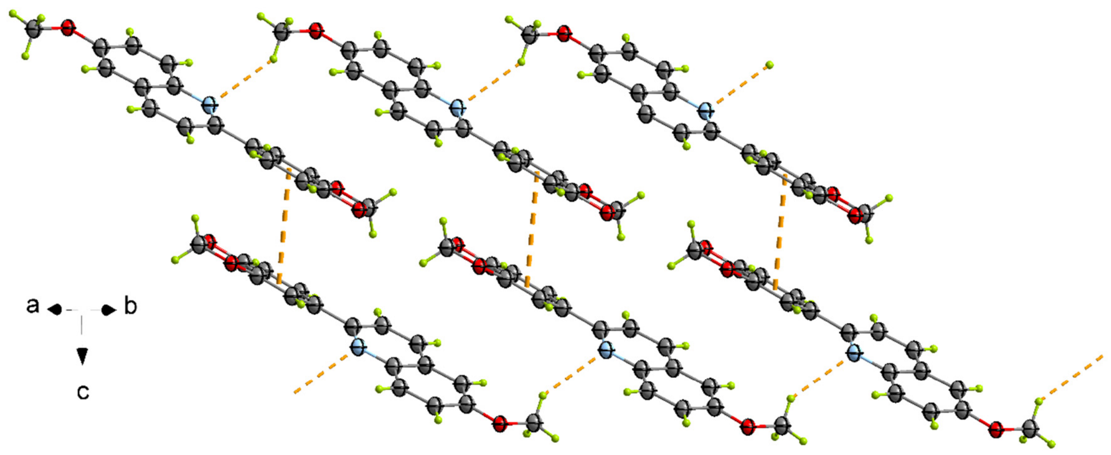

Figure S16. *Perspective view of* ***EP03*** *showing the weak C(sp³)–H···N chain [symmetry code (iv) x+1, y−1, z] propagating along the [1−10] direction, and the secondary p···p stacking interactions between benzodioxole rings [symmetry code (v) −x+2, −y+1, −z+1] that connect these chains into layers. Relevant contacts are shown as dashed lines.*

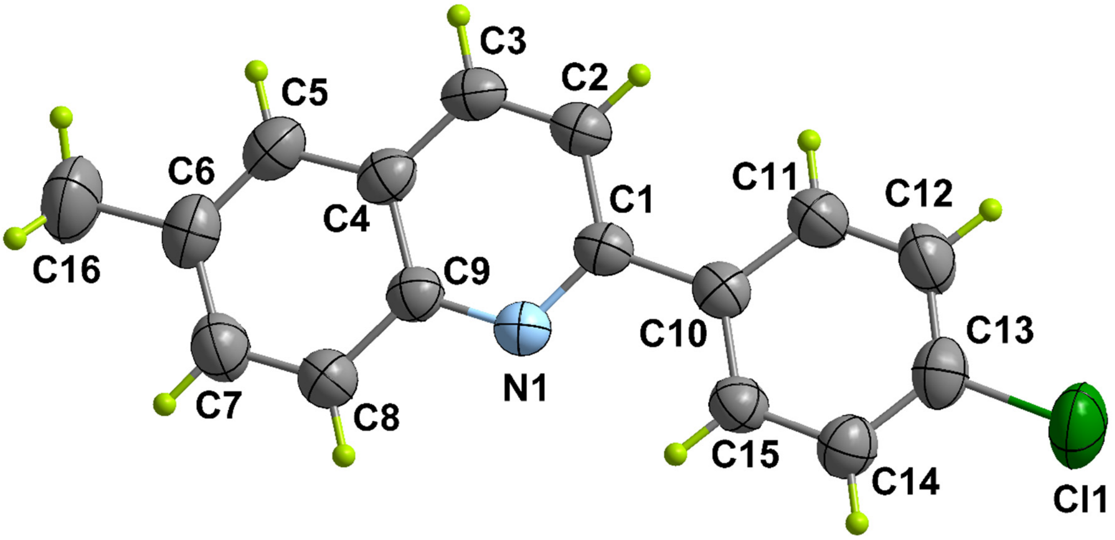

Figure S17. *View of the asymmetric unit of **EP04** with the numbering scheme of molecular structure. Atomic displacement ellipsoids are drawn at the 50% probability level.*

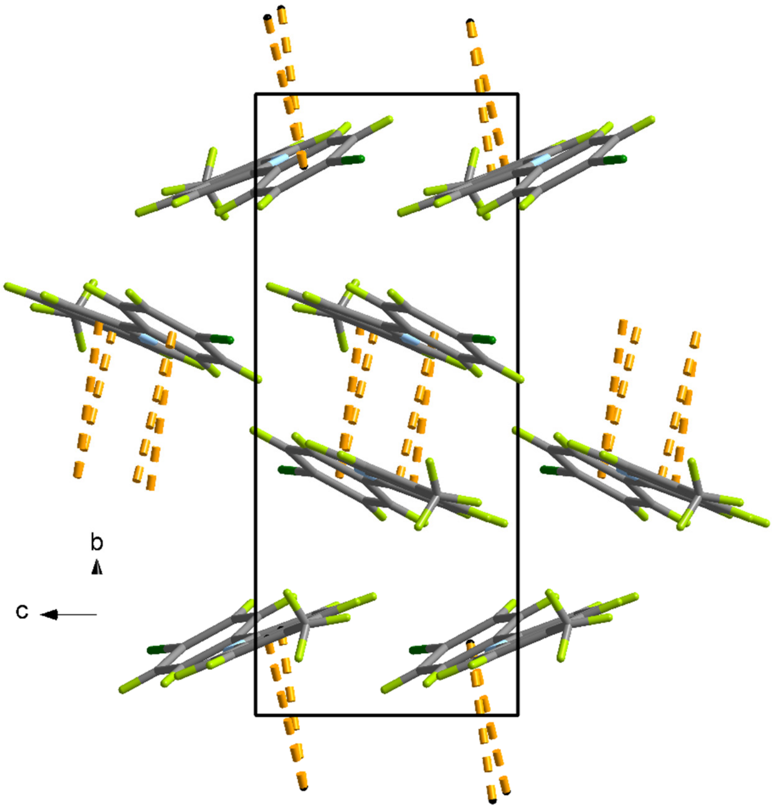

Figure S18. *Perspective view of* ***EP04*** *showing the two independent p···p stacking contacts between the pendant 4-chlorophenyl ring and both rings of a symmetry-related quinoline system [symmetry code (vi) −x+1, −y+1, −z+1], generating discrete centrosymmetric dimers. Relevant contacts are shown as dashed lines.*

Table S9. *Crystal data, data collection, and structure refinement for* ***EP01, EP02, EP03 and EP04*** *compounds.*

**Compound**	**EP01**	**EP02**	**EP03**	**EP04**
Formula	$C_{20}H_{19}NO_2$	$C_{19}H_{16}FNO_3$	$C_{17}H_{13}NO_3$	$C_{16}H_{12}ClN$
FW (mol/g)	305.36	325.33	279.28	253.72
Temperature (K)	296.15	296.15	296.15	296.15
Crystal system	Orthorhombic	Monoclinic	Triclinic	Monoclinic
Space group	$P2_12_12_1$	$P2_1/n$	P-1	$P2_1/c$
a (Å)	7.7122(4)	6.916(2)	6.4907(5)	13.8038(8)
b (Å)	10.7283(6)	17.267(7)	7.1686(5)	14.717(1)
c (Å)	19.5344(9)	13.285(6)	15.282(1)	6.2330(4)
a (°)	90	90	77.653(3)	90
b (°)	90	101.59(1)	80.052(3)	95.335(2)
g (°)	90	90	74.827(3)	90
Volume (Å^3)	1616.2(1)	1554.2(1)	665.22(8)	1260.7(1)
Z	4	4	2	4
r_{calc} g (cm^3)	1.255	1.390	1.394	1.337
m (mm^{-1})	0.081	0.102	0.096	0.282

F(000)	648.0	680.0	292.0	528.0
Crystal size (mm^3)	0.24×0.08x0.02	0.08×0.04×0.03	0.14×0.13×0.08	0.38×0.12×0.05
Radiation	Mo Ka (l= 0.71073)	Mo Ka (l= 0.71073)	Mo Ka (l= 0.71073)	Mo Ka (l= 0.71073)
2q range for data collection (°)	4.17 to 52.832	3.918 to 50.972	5.500 to 50.716	4.054 to 50.71
Index ranges	-9 ≤ h ≤ 9<br>-13 ≤ k ≤ 13<br>-24 ≤ l ≤ 24	-8 ≤ h ≤ 7<br>-20 ≤ k ≤ 20<br>-16 ≤ l ≤ 15	-7 ≤ h ≤ 7<br>-8 ≤ k ≤ 8<br>-18 ≤ l ≤ 18	-16 ≤ h ≤ 16<br>-17 ≤ k ≤ 17<br>-7 ≤ l ≤ 7
Reflections collected	27812	33607	17622	20701
Independent reflections	3322 [R_{int} = 0.1013]	2864 [R_{int} = 0.1271]	2421 [R_{int} = 0.0559]	2308 [R_{int} = 0.0489]
Data/restraints /parameters	3322/0/210	2864/0/221	2421/0/191	2308/0/164
Goodness-of-fit on F^2	1.039	1.061	1.047	1.064
Final R indexes I>=2s (I) [all data]	R^1 = 0.0434 / wR^2 = 0.0981 [R^1 = 0.0724 / wR^2 = 0.1132]	R^1 = 0.0493, wR^2= 0.1128 [R^1 = 0.0907, wR^2 = 0.1374]	R^1 = 0.0428 / wR^2= 0.1143 [R^1 = 0.0599 / wR^2 = 0.1270]	R^1 = 0.0407 / wR^2= 0.1160 [R^1 = 0.0477 / wR^2 = 0.1224]
Largest diff. peak/hole / e $Å^{-3}$	0.21/-0.15	0.17/-0.16	0.137/-0.126	0.202/-0.235

Table S10. *Geometric parameters of the relevant intermolecular interactions in* ***EP01*** *–* ***EP04****.*

**Comp.**	**Interaction**	**H···A (Å)**	**D···A (Å)**	**D–H···A (°)**	**Cg···Cg (Å)**	**Offset angle (°)**	**Sym. code**
**EP01**	p···p	–	–	–	3.9516(1)	28.03(8)	(i)
**EP02**	O3–H3···N1	2.05(4)	2.917(3)	171.4(3)	–	–	(ii)
**EP02**	p···p	–	–	–	3.790(1)	21.42(7)	(iii)
**EP03**	C17–H17A···N1	2.695	3.515	143.7	–	–	(iv)
**EP03**	p···p	–	–	–	3.955	25.23	(v)
**EP04**	p···p	–	–	–	3.7992(2)	17.95	(vi)
**EP04**	p···p	–	–	–	3.9949(2)	25.92	(vi)

Symmetry codes: (i) *x*, *y*–1, *z*; (ii) *x*+1/2, −*y*+3/2, *z*–1/2; (iii) −*x*+1, −*y*+1, −*z*+1; (iv) *x*+1, *y*–1, *z*; (v) −*x*+2, −*y*+1, −*z*+1; (vi) −*x*+1, −*y*+1, −*z*+1.
